# Genome-Wide Tissue-Specific Occupancy of the Hox Protein Ultrabithorax and Hox Cofactor Homothorax in *Drosophila*

**DOI:** 10.1371/journal.pone.0014686

**Published:** 2011-04-05

**Authors:** Matthew Slattery, Lijia Ma, Nicolas Négre, Kevin P. White, Richard S. Mann

**Affiliations:** 1 Department of Biochemistry and Molecular Biophysics, Columbia University, New York, New York, United States of America; 2 Department of Human Genetics, Department of Ecology and Evolution, Institute for Genomics and Systems Biology, University of Chicago, Chicago, Illinois, United States of America; Georgia Institute of Technology, United States of America

## Abstract

The Hox genes are responsible for generating morphological diversity along the anterior-posterior axis during animal development. The *Drosophila* Hox gene *Ultrabithorax* (*Ubx*), for example, is required for specifying the identity of the third thoracic (T3) segment of the adult, which includes the dorsal haltere, an appendage required for flight, and the ventral T3 leg. *Ubx* mutants show homeotic transformations of the T3 leg towards the identity of the T2 leg and the haltere towards the wing. All Hox genes, including *Ubx*, encode homeodomain containing transcription factors, raising the question of what target genes *Ubx* regulates to generate these adult structures. To address this question, we carried out whole genome ChIP-chip studies to identify all of the Ubx bound regions in the haltere and T3 leg imaginal discs, which are the precursors to these adult structures. In addition, we used ChIP-chip to identify the sites bound by the Hox cofactor, Homothorax (Hth). In contrast to previous ChIP-chip studies carried out in *Drosophila* embryos, these binding studies reveal that there is a remarkable amount of tissue- and transcription factor-specific binding. Analyses of the putative target genes bound and regulated by these factors suggest that Ubx regulates many downstream transcription factors and developmental pathways in the haltere and T3 leg. Finally, we discovered additional DNA sequence motifs that in some cases are specific for individual data sets, arguing that Ubx and/or Hth work together with many regionally expressed transcription factors to execute their functions. Together, these data provide the first whole-genome analysis of the binding sites and target genes regulated by Ubx to specify the morphologies of the adult T3 segment of the fly.

## Introduction

Hox genes encode an evolutionarily conserved set of homeodomain-containing transcriptional regulators that play critical roles in the development of all metazoans. Although first discovered in *Drosophila* because of their role in anterior (A)-posterior (P) axial patterning, these genes are now known to assign morphological identities along the AP axes in both vertebrates and invertebrates [1], [2], [3],[4]. However, their importance in animal development extends far beyond AP patterning, and includes functions from stem cell maintenance to motor neuron specification and leukemogenesis [5], [6], [7], [8].

To execute their various functions, Hox proteins regulate the transcription of many types of target genes, of which only a handful are known [9], [10]. Some estimates of the number and types of Hox target genes have come from expression profiling experiments, in cell lines or embryos in which Hox expression was manipulated. For example, ectopic expression of six of the eight Hox genes in *Drosophila* embryos led to the altered expression of approximately 1500 genes, suggesting that ∼10% of all genes in the fly genome could be Hox-regulated [11]. For nearly a third of these genes, expression was altered by multiple Hox factors, suggesting that many targets are regulated by more than one Hox gene. One caveat to studies such as this is that they rely on ectopic expression, which may induce non-physiological gene regulation and thus result in an inflated estimate of the number of Hox target genes. In an alternative approach, the transcriptomes of two homologous tissues, the *Drosophila* wing and haltere imaginal discs, which give rise to the dorsal regions of the second (T2) and third (T3) thoracic segments, respectively, were compared [12]. All of the differences between these two tissues are due entirely to a single Hox gene, *Ultrabithorax* (*Ubx*), which is expressed in one (the haltere, a balancing organ required for flight) but not the other (the wing). Thus, a comparison between the genes expressed in these two wild type tissues has the potential to identify genes that are either activated or repressed by Ubx. In this study by Hersh *et al.*, the number of differentially expressed genes was less than 500, suggesting a much more limited number of targets. However, it is likely that this number is an underestimate, because expression profiling studies will miss target genes whose expression patterns are altered by Hox regulation, but are still expressed, on average, at similar levels in both tissues. In addition, any approach that relies on expression profiling cannot distinguish between directly and indirectly regulated target genes.

A third method to identify Hox target genes is to take a candidate approach [13]. Based on the observation that the haltere imaginal disc has ∼3-fold fewer cells than the wing imaginal disc, Ubx is expected to regulate genes that reduce the amount of cell proliferation in the haltere. Consistent with this idea, Ubx was found to regulate several aspects of the Decapentaplegic (Dpp) morphogen signaling pathway, which is required for imaginal disc growth [14], [15], [16], [17]. Interestingly, the expression patterns of these target genes, such as the Dpp receptor *thickveins* (*tkv*) and the proteoglycan *dally*, differ in the haltere compared to the wing in a manner that restricts the diffusion of Dpp in the haltere, thus limiting the extent of pathway activation. These observations underscore the idea that instead of simply turning genes on or off, Hox genes contribute to the regulation of spatial patterns of gene expression. Although such a candidate approach is good at identifying biologically relevant target genes, the approach is limited in scope and typically cannot discriminate between direct and indirect regulation.

Hox proteins typically bind to degenerate AT-rich DNA sequences [18]. The monomer binding site for Ubx, for example, is TAAT[tg]G [19], [20]. Based on this low degree of sequence specificity, Ubx monomer binding sites are predicted to occur several times per kilobase in eukaryotic genomes. The large number of potential Hox binding sites, on average more than ten per transcription unit, contrasts with the highly gene-specific regulation Hox proteins, and Ubx in particular, carry out *in vivo* [9], [11], [12]. Complicating the Hox specificity problem is that this family of proteins, which are encoded by eight Hox paralogs in *Drosophila* and 39 Hox genes in vertebrates, all bind to very similar DNA sequences via identical DNA-contacting residues in their homeodomains [19], [20], [21], [22]. One way in which Hox proteins achieve a higher degree of DNA binding specificity is to bind cooperatively with cofactors. One such cofactor is a heterodimer composed of Extradenticle (Exd; Pbx in vertebrates) and its binding partner Homothorax (Hth; Meis in vertebrates), both homeodomain proteins [22], [23], [24]. Together, Exd-Hth bind cooperatively with Hox proteins, allowing them to recognize structural features of the DNA that cannot be read in the absence of these cofactors [25]. However, Hox proteins do not always bind to biologically relevant binding sites with Exd and Hth, and they are also capable of regulating target genes without the help of these cofactors [26], [27], [28]. In the haltere imaginal disc, for example, Exd and Hth are only available to bind DNA with Ubx in the cells that will give rise to the proximal regions of this appendage and body wall [29]. Therefore, in cells that will give rise to most of the appendage, Ubx must regulate target genes without the help of these cofactors. Whether there are other Hox cofactors in this region of the imaginal disc remains an open question.

The functions that Hox proteins execute, and thus the target genes they regulate, must be dependent on the cellular context. *Ubx*, for example, is not only expressed in haltere imaginal discs, but is also expressed in the imaginal discs that will give rise to the T3 legs. In the absence of *Ubx*, the identity of the T3 leg is transformed into the identity of the T2 leg; analogously, the identity of the haltere is transformed into that of the wing, also a T2 structure [2], [30], [31]. The difference between these two pairs of homologous tissues is that the wing/haltere pair gives rise to dorsal regions of the fly and express dorsal identity genes, such as *pannier*, *mirror,* and *vestigial*, while the leg discs give rise to ventral regions of the fly and express ventral identity genes, such as *Sp1*, *Distalless*, and *dachshund* [32], [33], [34]. Thus, the expectation is that Hox proteins such as Ubx regulate their target genes in conjunction with either dorsal or ventral identity genes. In addition, Hox proteins also collaborate with transcription factors downstream of signaling pathways, such as Dpp and Wingless (Wg) [3], [22], [35]. Differences along the proximo-distal (PD) axis are also likely to influence Hox regulation; as in the haltere imaginal disc, Exd and Hth are only expressed or nuclear in the cells of the leg imaginal discs that will give rise to the proximal regions of these appendages and ventral body wall [29]. Thus, Ubx cannot use these cofactors to regulate its target genes in the cells that will give rise to leg segments distal to the trochanter.

**Figure 1:**
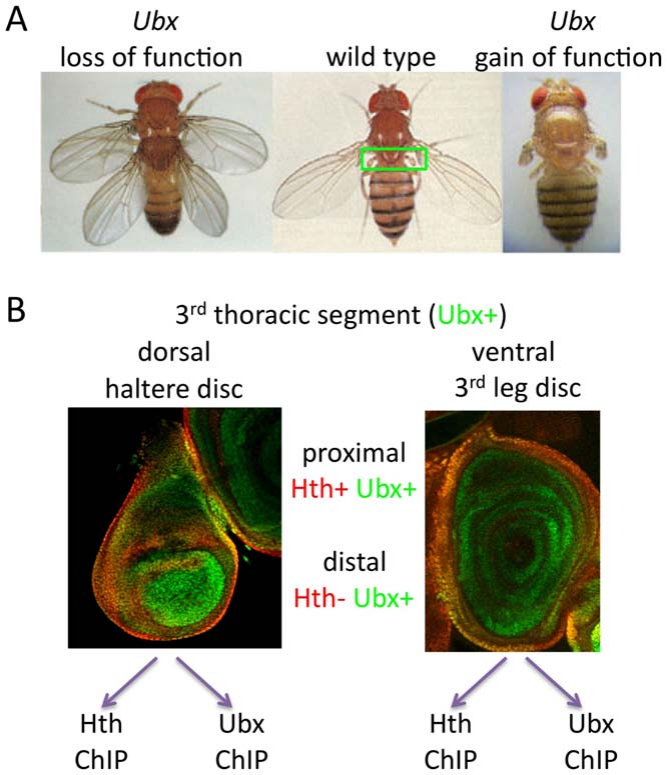
Overview of ChIP-chip analysis. A) *Ubx* is responsible for all of the differences between the second (T2) and third (T3) thoracic segments. *Ubx* loss-of-function mutations transform halteres (green box, center panel) into wings (left panel); *Ubx* gain-of-function in T2 transforms the wings into halteres (right panel). B) In this study, tissue-specific chromatin immunoprecipitation was performed with pure populations of haltere or T3 leg imaginal discs, both of which express Ubx in all cells (green) and express Hth (red) in the subset of cells that will give rise to proximal appendages and body wall.

In the work described here, we use chromatin immunoprecipation (ChIP) and whole genome, high density tiling arrays to identify the sequences bound by Ubx and Hth in both the T3 leg and haltere imaginal discs (Figure 1). Unlike ChIP experiments carried out using embryos, which are composed of many different cell types, these experiments analyze the binding of these transcription factors in two tissues with much less cell-type complexity. Because both the haltere and T3 leg express *Ubx* in all cells, and require this Hox gene for their unique identities, a comparison between the binding sites in these two tissues has the potential to identify haltere- and leg-specific binding events. Further, by also analyzing Hth binding, which is a surrogate for Exd+Hth binding, we can distinguish between Ubx-Exd-Hth binding events (which can only occur in cells that will give rise to proximal structures) from Exd-Hth-independent Ubx binding (which can occur in all cells). To our knowledge, these experiments provide one of the first *in vivo* tissue-specific comparisons between genome-wide ChIP-chip data sets. In contrast to whole embryo ChIP-chip studies, the data reveal both tissue-specific and non-specific binding by these factors. Together, these comparisons lead to a comprehensive view of the target genes being regulated by Ubx, and provide a valuable platform for exploring Hox specificity and the network of genes required to generate these two regions of the adult fly.

## Results and Discussion

Ubx is required to establish the fates of the T3 segment of the adult fly, which is comprised of both dorsal body structures, including the haltere, and ventral body structures, including the T3 leg (Figure 1). As a first step towards identifying the genes that Ubx regulates to generate these two parts of the adult fly, we identified where Ubx is bound in the wild type T3 leg and haltere imaginal discs using chromatin immunoprecipitation (ChIP). In addition, we also used ChIP to identify Hth-bound regions (Figure 1). In both cases, we used polyclonal antibodies raised against native Ubx or Hth proteins to immunoprecipitate bound chromatin fragments from wild type T3 leg or haltere imaginal discs. Immunoprecipitated fragments were labeled and used to probe high-density, whole-genome tiling arrays (Methods). In addition to several lines of supporting evidence presented below, the haltere data sets are positively correlated with those independently generated by S. W. Choo, S. Russell, and R. White (personal communication) using a different set of reagents, arguing that they represent true binding events. A comparison with these independently obtained haltere Ubx and Hth binding data, described in Methods, is shown in Supplementary Figure S1, along with tables of high confidence target genes that are shared between both data sets (Supplementary Tables S1 and S2). We use our data to address two general questions: What are the target genes bound by Ubx (with and without Hth) in the T3 leg and haltere imaginal discs? Second, what can we learn about tissue-specific binding for Ubx (with and without Hth)?

### Overview of Ubx and Hth binding events

Table 1 provides an overview of the number of binding events identified in each of these ChIP experiments. We define a bound region as a peak that is identified as being in the top 5% of p-values using Tiling Analysis Software (Affymetrix) and overlapping a peak called at a 5% false discovery rate using model-based analysis of tiling arrays (MAT, see Methods for more details). Although the difference in binding events for Hth in the leg and haltere is less than 2-fold (∼1000 and ∼600, respectively), there are ∼5-fold more binding events for Ubx in the haltere compared to the leg (∼4600 and ∼900, respectively). The large difference in Ubx binding sites identified in these two tissues may reflect the fact that there are many more morphological differences between the haltere and wing compared to the T3 and T2 legs.

**Table 1:** Ubx and Hth binding overview.

	Factor	Total binding (#)	Overlap with other tissue (%)	Overlap with other factor (%)
**Haltere**	**Ubx**	4590	15.9	7.5
	**Hth**	559	72.1	58.9
	**Ubx+Hth**	345	22	NA
**Leg**	**Ubx**	867	84.4	20.4
	**Hth**	1062	37.9	16
	**Ubx+Hth**	177	42.9	NA
**Haltere, not leg**	**Ubx**	3858	0	2.3
	**Hth**	164	0	51.2
	**Ubx+Hth**	89	0	NA
**Leg, not haltere**	**Ubx**	126	0	14.3
	**Hth**	659	0	2.7
	**Ubx+Hth**	18	0	NA

These numbers also indicate that there is a large amount of specificity in Ubx binding that cannot be accounted for simply by its preference to bind its monomer binding site, TAAT[t/g]G. Not only is a small subset of the total number of TAAT[t/g]G sites occupied in either tissue (see below), the number and type of binding events differ in a tissue-specific manner. For example, even though there are ∼5-fold fewer Ubx binding events in the T3 leg compared to the haltere, ∼16% of these are not observed in the haltere. We return to the question of tissue specificity below.

### Target gene and GO analysis

**Figure 2:**
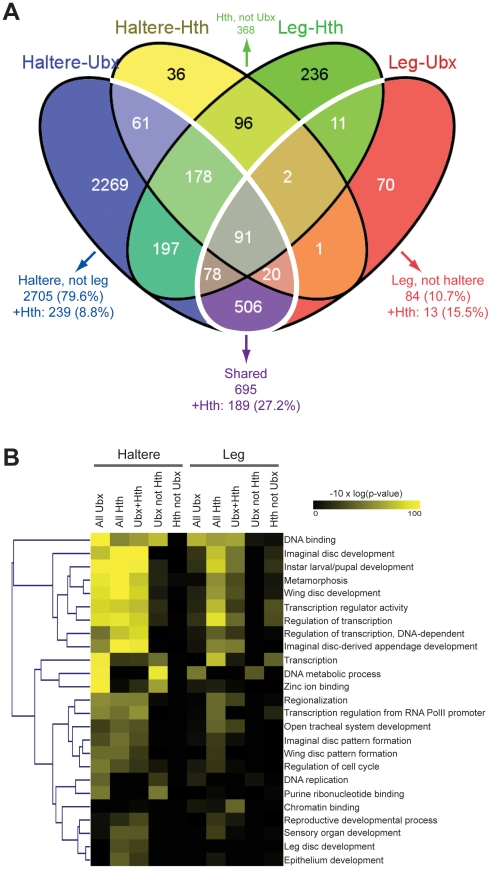
Ubx and Hth target gene classifications. A) Venn diagram comparing genes called as Ubx targets in haltere or T3 leg imaginal discs to genes called as Hth targets in those tissues. B) Overrepresentation of Gene Ontology biological processes or molecular functions among various subsets Ubx and Hth target genes. For the indicated tissue, columns represent enriched GO categories for all genes targeted by Ubx (All Ubx), all Hth targets (All Hth), genes targeted by both Ubx and Hth (Ubx+Hth), genes targeted by Ubx but not targeted by Hth (Ubx not Hth), and genes targeted by Hth but not targeted by Ubx (Hth not Ubx).

Although only a subset of the binding events identified in these ChIP experiments is likely to be functional, it is nevertheless of interest to ask how many and what types of target genes Ubx is binding to in these two tissues. To explore the genes targeted by Ubx, each Ubx or Hth ChIP peak was assigned a target gene based on the nearest transcription start site (see Methods for details). Using this method of calling target genes, Ubx targets a total of 3400 gene in the haltere and 779 genes in the leg, while Hth targets 485 genes in the haltere and 889 in the leg. A breakdown of the numbers of target genes is shown in Figure 2A. Consistent with the apparent specificity observed in the binding site analysis, there is also a significant amount of specificity in the genes that Ubx and Hth are potentially regulating (Figure 2A). For example, about 80% of the called Ubx target genes in the haltere are not target genes in the leg, and about 11% of the called Ubx target genes in the leg are not target genes in the haltere. A similar picture emerges for putative Hth target genes: ∼20% of called Hth target genes in the haltere are not targets in the leg, and about 54% of the called Hth target genes in the leg are not targets in the haltere.

The predominant Gene Ontology (GO) categories for Ubx target genes, in both the leg and the haltere, are those involved in transcription, such as “DNA binding” and “Transcription”, indicating that Ubx regulates the expression of a large number of subordinate transcription factors in both tissues (Figure 2B). This picture remains largely the same for genes that have both Ubx and Hth inputs, and is also true for targets that have Hth, but not Ubx binding. Other highly significant GO categories are “Imaginal disc development”, “Instar larval/pupal development”, and “Metamorphosis”, consistent with the source of the tissues used for these experiments. Two additional noteworthy categories are “Wing disc development” and “Wing disc pattern formation”, both of which are more significantly associated with the haltere data sets compared to the leg data sets. This difference makes sense given that the haltere is a modified wing, and implies that, in the haltere, Ubx is more likely to regulate genes involved in wing development compared to the T3 leg. Overall, the consistency between these GO categories and the expected functions of Ubx in imaginal disc development suggests that many of the Ubx target genes identified by these ChIP experiments are biologically relevant targets. Interestingly, Ubx target genes that contain multiple, distinct Ubx peaks tend to be enriched for genes encoding key developmental regulators (p<10^−5^ for both “DNA binding” and “imaginal disc development” in the haltere); Ubx target genes with only one peak are more enriched for regulators of metabolism and the cell cycle (not shown). Examination of the Ubx-bound genes in more detail (Supplementary Table S5) reveals that Ubx is bound to genes involved in many different cellular pathways that execute a wide variety of functions. For example, in the Ubx-bound leg, not haltere data set (84 total genes), there are genes involved in mRNA processing (e.g. *hiiragi* (FBgn0015949) and *Repressor splicing factor 1* (FBgn0011305)) and mitosis (e.g. *Sak kinase* (FBgn0026371) and *Septin 4* (FBgn0259923)). In the Ubx-bound haltere, not leg data set (2705 total genes) there are genes involved in bristle morphogenesis (e.g. *pawn* (FBgn0003174) and *forked* (FBgn0000630)) and cell cycle control (e.g. *decapo* (FBgn0010316) and *Cyclin E* (FBgn0010382)). Genes involved in *Notch* signaling (e.g. *fringe* (FBgn0011591), *numb* (FBgn0002973), and *Tace* (FBgn0039734) are also specifically enriched in this data set. The wide range of functions represented by these gene lists suggests that *Ubx* is not only regulating transcription factors, but also genes involved in many aspects of the development of the leg and haltere, including terminal differentiation genes.

**Figure 3:**
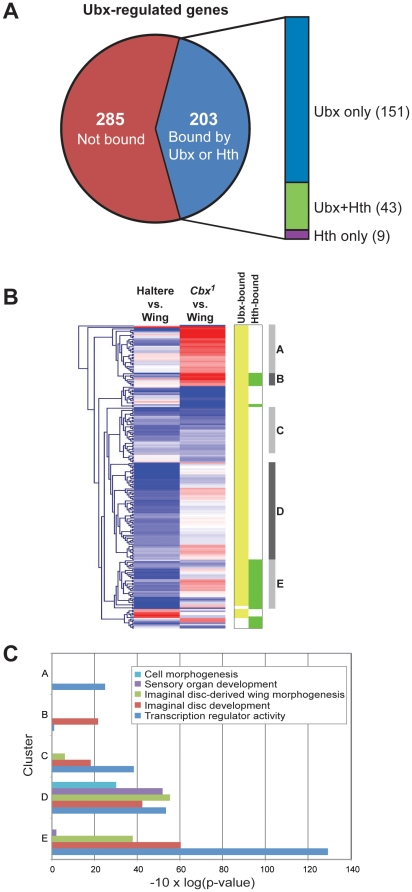
Ubx-regulated gene expression and Ubx occupancy. A) Fraction of Ubx-responsive genes based on gene expression profiling that is identified as Ubx and/or Hth target genes in the haltere using ChIP-chip. Ubx-responsive genes are those called as up- or downregulated in haltere discs or wing discs ectopically expressing *Ubx* (*Cbx^1^* mutant discs) as compared to wild type wing discs (expression data from [12]). B) Heatmap representing expression levels in the haltere or *Cbx^1^* wing relative to wild type wing. Higher expression in haltere or *Cbx^1^* is red; lower expression in haltere or *Cbx^1^* is blue. Genes are hierarchically clustered. Two columns to the right of the heatmap indicate binding by Ubx (light green) or Hth (dark green) at a given gene locus. Five clusters (indicated on the far right) representing unique binding and expression profiles were found to found to be enriched for one or more GO categories. Cluster A consists of genes upregulated by ectopic *Ubx* expression in the wing that are bound by Ubx, whereas cluster B contains genes with the same expression pattern that are bound by Ubx and Hth. Cluster C consists of Ubx-bound genes that are expressed at lower levels in the haltere or upon ectopic expression of *Ubx* in the wing. Clusters D and E consist of genes that are expressed at lower levels in the haltere relative to the wing; only Ubx binds genes in cluster D, whereas Ubx and Hth bind those in cluster E. C) Enrichment of Gene Ontology biological process or molecular function categories among clusters A through E (described above).

Additional validation of these data came from comparing the genes identified in the ChIP-chip experiments with those identified in expression profiling experiments. Of the 488 genes that were regulated by Ubx in haltere–wing transcriptome comparisons [12], 191 were bound by Ubx in the haltere which is a statistically significant enrichment (p<10^−5^; Figure 3A and Supplementary Table S6). In the published transcriptome experiments, two pair wise comparisons were made: wild type wing discs versus wild type haltere discs and wild type wing discs versus *Cbx^1^* wing discs, which are transformed towards haltere due to misexpression of *Ubx* [2]. Interestingly, clustering the genes according to their behavior in these two expression profiling experiments leads in some cases to the enrichment of distinct GO categories (Figure 3B,C). For example, for genes that are highly expressed in *Cbx^1^* wing discs, Ubx+Hth targeted genes tend to be involved in imaginal disc development, while Ubx (and not Hth) targeted genes tend to encode transcription factors (clusters B and A, respectively, in Figure 3C). These observations not only help to validate the ChIP-chip targets defined here, but they also reveal a previous unknown degree of specificity in gene regulation by Ubx and Hth.

### Examples of Ubx-bound genes: transcription factors

Another way to assess the validity of the target genes identified by these ChIP-chip experiments is to examine individual genes that are predicted to be regulated by Ubx based on genetic criteria. A complete list of the target genes, organized according to the tissue-specific binding of Ubx and Hth, is provided in Supplementary Table S5. Because genes encoding transcription factors comprise one of the largest groups Ubx-bound genes, we focus in this section on a subset of these genes.

**Figure 4:**
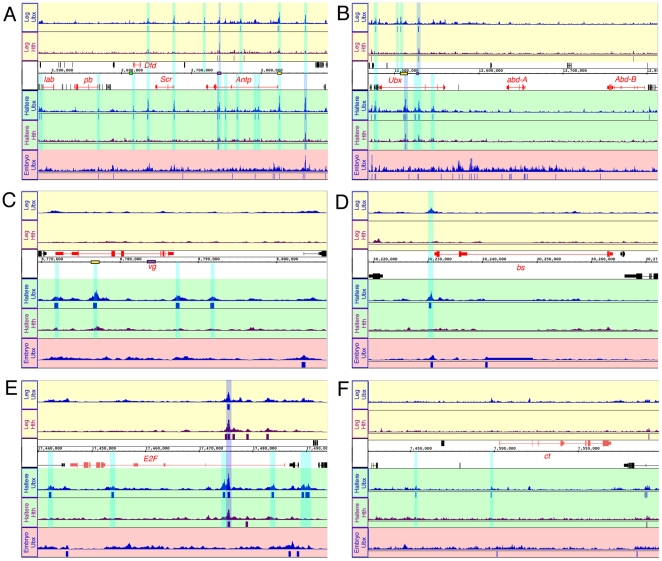
Examples of Ubx bound genes encoding transcription factors. Ubx and Hth binding profiles in the T3 leg and haltere imaginal discs at the Antennapedia Complex (ANT-C) (A), the Bithorax Complex (BX-C) (B), *vestigial (vg)* (C), *blistered/DSRF (bs)* (D), *E2F* (E), and *cut (ct)* (F). Regions called as bound by Ubx but not Hth are highlighted in turquoise, and regions bound by both Ubx and Hth are highlighted in blue. Selected known enhancers are represented as boxes along the chromosomal map. For (A), the Dfd EAE element is represented with a green box, Antp P2 with a purple box, and Antp P1 with yellow box. For (B), the *Ubx* abx and bx1 enhancers are represented by yellow and purple boxes, respectively. For (C), the *vg* boundary and quadrant enhancers are represented by yellow and purple boxes, respectively. A track showing Ubx binding in 0–12 hr embryos is shown at the bottom of each panel for comparison. Tracks represent −10×log(p-value) as generated by TAS (methods).

One set of genes that are transcriptionally regulated by Hox proteins is the Hox genes, themselves. The general rule from genetic studies is that more posteriorly expressed Hox genes have the ability to repress more anteriorly expressed Hox genes [36], [37], [38], [39]. Ubx, for example, can repress *Antennapedia* (*Antp*), *Sex combs reduced* (*Scr*), and the head-determining Hox genes *Deformed* (*Dfd*) and *labial* (*lab*), but not the more posteriorly expressed abdominal Hox genes, *abdominal-A* (*abd-A*) and *Abdominal-B* (*Abd-B*). Ubx is also known to negatively autoregulate its own transcription [40], [41], [42]. However, it was not known if any of this regulation is direct. Strikingly, in both the T3 leg and haltere, we observe Ubx or Ubx+Hth binding near *lab*, *proboscipedia* (*pb*), *Dfd*, *Scr*, *Antp*, and *Ubx*, but not in the vicinity of *abd-A* and *Abd-B* (Figure 4A,B). These data suggest that Ubx's ability to repress more anterior Hox genes is due to direct binding to these genes. Further, these data reveal that in both the T3 leg and haltere imaginal discs Ubx is not significantly bound to *abd-A* and *Abd-B*, which includes more than 200 kb of genomic DNA, providing a dramatic example of Ubx binding specificity matching Ubx functional specificity. It is also worth noting that Ubx appears to bind near *abd-A* during embryonic development (Figure 4B). The fact that this binding disappears in the imaginal discs may indicate that the there is tissue-specific Ubx binding to *abd-A* in the embryo (e.g. in the CNS or elsewhere) that is not there in the leg or haltere disc. It is also possible that the loss of this binding event in the imaginal discs is due to developmental changes in chromatin structure, possibly via Polycomb-mediated silencing, play a role in the specificity of Ubx binding to Hox loci.

*vestigial* (*vg*) encodes a transcription factor that plays a central role in the development of both dorsal appendages (the wing and haltere), but is not required for the development of ventral structures such as the legs. *vg* has a different expression pattern in the haltere compared to the wing, suggesting regulation by *Ubx* [13]. Consistent with both of these observations, we find four Ubx binding events in the vicinity of *vg* in the haltere, but none in the T3 leg (Figure 4C). Ubx binding in the haltere, but not T3 leg, is also observed at *cut*, which encodes a transcription factor required for neurogenesis at the wing margin, and is repressed in the haltere [13] (Figure 4F). Ubx is also bound to *blistered* (*bs*, also known as *DSRF*), which is required for wing vein formation and is differentially expressed in the wing compared to the haltere [13]. In this case, a strong Ubx binding event is observed in the haltere, which has no veins, suggesting direct repression by Ubx. The same binding event is also observed in the T3 leg, although with apparently weaker affinity (Figure 4D).

Finally, we highlight *E2F,* which encodes a transcription factor that positively regulates many genes required for cell cycle progression [43]. Given the difference in cell number between the haltere and wing, it is of interest that there are five Ubx binding sites in the haltere. One of these, which also shows Hth binding, is also observed in the T3 leg, suggesting that this gene may be regulated by Ubx+Hth in proximal T3 leg disc cells (Figure 4E).

### Examples of Ubx-bound genes: signaling pathways

**Figure 5:**
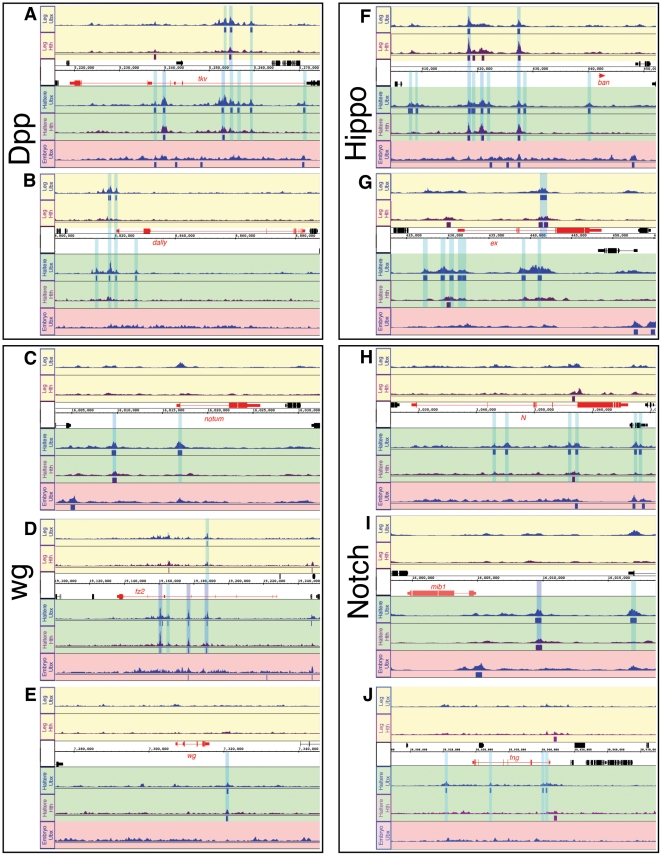
Examples of Ubx bound genes encoding signaling pathway components. Ubx and Hth binding profiles at the Dpp pathway components *thickveins (tkv)* (A), *dally* (B), and *notum* (C); at the Wg pathway components *frizzled 2 (fz2)* (D), and *wingless (wg)* (E); at the Hippo pathway components *bantam (ban)* (F) and *expanded (ex)* (G); and at the Notch pathway components *Notch (N)* (H), *mind bomb 1* (*mib1)* (I), and *fringe (fng)* (J). Color scheme and tracks are as described in Figure 4.

After genes involved in transcriptional regulation, the most significant GO categories for Ubx-bound genes are those involved in development, including cell-cell signaling. Previous work suggested that signaling pathway genes were targets of *Ubx* in the haltere, although it was not determined if any of this regulation is direct. In Figure 5, we highlight seven genes that are integral components of or influence the activities of the Notch (N), Dpp, Wingless (Wg), or Hippo signaling pathways. Of particular interest are genes in the Dpp pathway, due to its role in haltere size control [16]. Consistently, we find direct binding of Ubx to the Dpp receptor *thickveins* (*tkv*) and the glypican *dally* (Figure 5A,B). In the Wg pathway, we observe direct binding of Ubx in the haltere to the Wg receptor, *frizzled2* (*fz2*), to *wg*, itself, and *notum*, which encodes a secreted hydrolase that also modulates Dpp and Hedgehog signaling pathways [44], [45], [46] (Figure 5C–E). In both the haltere and T3 leg, we also observe Ubx binding to two genes in the Hippo signaling pathway, the microRNA *bantam* (*ban*) and *expanded* (*ex*), which is required for cell proliferation and survival [47], [48] (Figure 5F,G). Finally, Ubx is bound to *Notch* (*N*), *mind bomb 1* (*mib1*), and to *fringe (fng)* but only in the haltere, suggesting that the Notch pathway is being modulated in this tissue (Figure 5H,I). See Supplementary Figure S2 for additional examples of Ubx bound genes.

### Target gene validation by expression pattern analysis

**Figure 6:**
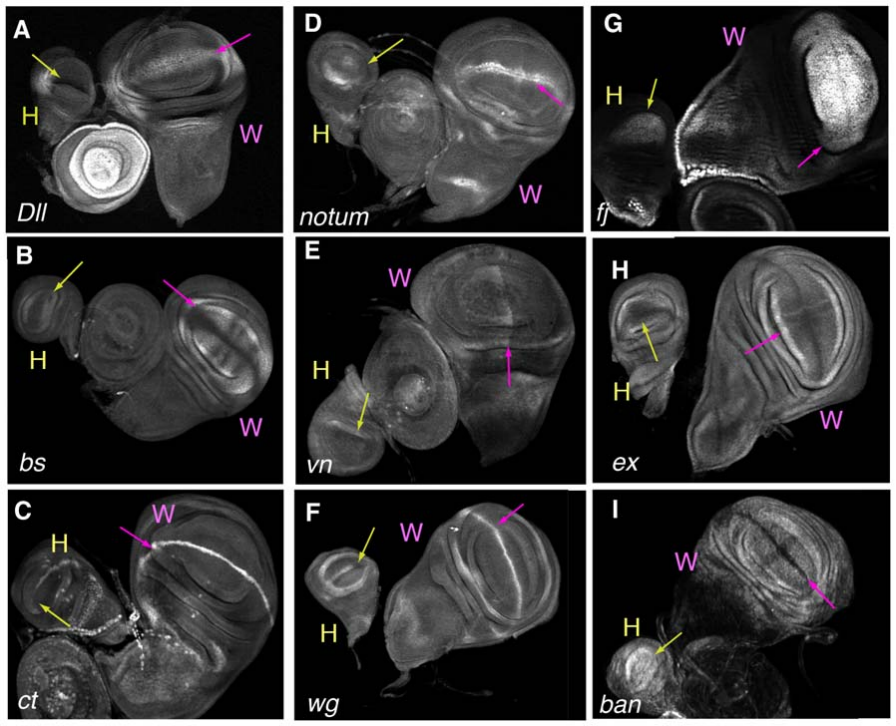
Expression analysis of predicted target genes in the haltere and wing. A) *Dll* expression along the DV axis of the wing is missing from the homologous region of the haltere. B) *bs*/*DSRF* is expressed in the pouch of the wing but is not expressed in the haltere. C) In comparison to the wing, *ct* expression along the DV axis is lower overall in the haltere, and is completely absent from the posterior compartment of the haltere. D) The stripe of *notum-lacZ* expression along the DV axis of the wing is almost entirely missing in the haltere, except for a low level of expression in the most medial region of this axis. E) *vn-lacZ* is expressed in the medial wing but repressed in the medial haltere. F) *wg-lacZ* expression along the DV axis is absent in the posterior compartment of the haltere. G) *fj-lacZ* expression is greatly reduced in the pouch of the haltere as compared to the pouch of the wing. H) *ex-lacZ* is expression is reduced in the pouch of the haltere. I) *ban-Gal4* is repressed along the AP and DV axes of the wing, but is expressed along these axes in the haltere.

As additional validation for the functionality of the observed Ubx binding sites, we extended previous expression analyses [12], [13], [14], [15], [17], [26], [27] and compared the expression patterns of a subset of Ubx-bound genes in the wild type haltere and wing imaginal discs, which are Ubx-expressing and –nonexpressing tissues, respectively. We tested a set of genes for which reagents (antibodies or enhancer traps) were readily available; while we did not select only genes known to be regulated by Ubx, we did not exclude these genes from analysis, either. In all cases examined, we were able to detect expression pattern differences in these two tissues, consistent with the idea that Ubx binding to these genes is functional (Figure 6). Interestingly, with only one exception, elements of the expression patterns present in the wing were missing in the haltere (e.g. *wg*, *notum*, and *Distalless* (*Dll*)), suggesting that Ubx is repressing these genes rather than activating them. The one exception is the microRNA gene *ban*, which is transcribed uniformly in the distal haltere. In contrast, in the homologous region of the wing *ban* is repressed at the dorsoventral and anteroposterior compartment boundaries (Figure 6I).

Together, these expression patterns not only help to validate the ChIP-chip data, but they also reinforce a common theme, which is that *Ubx* is modifying expression patterns, not simply turning genes on or off throughout the entire haltere. As *Ubx* is expressed in all haltere and T3 leg cells, these results imply that Ubx must collaborate with many other regionally expressed factors to modify each of these expression patterns in unique ways.

### Tissue specificity of Ubx and Hth binding

**Figure 7:**
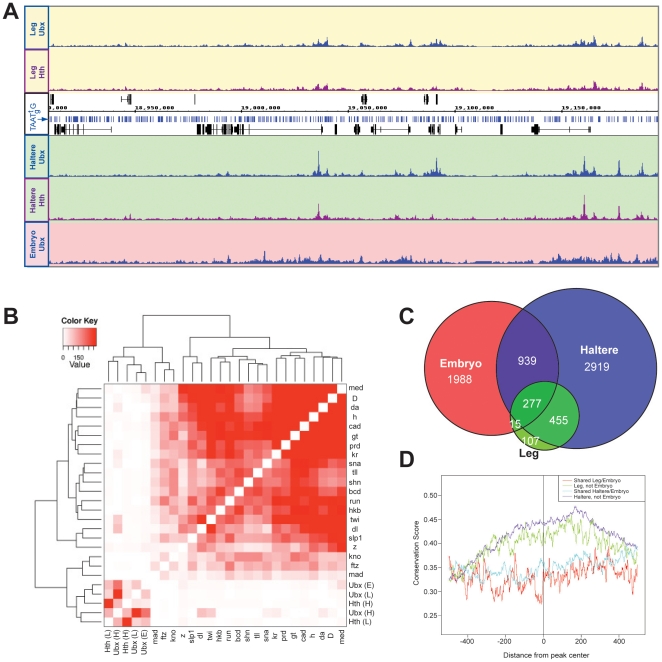
Tissue specificity of Ubx binding. A) Ubx and Hth binding profiles across approximately 300 kb of chromosome 3L (18,900,000–19,200,000). Sites matching the Ubx monomer consensus binding site TAAT[t/g]G are indicated as blue bars along the chromosomal map. B) Hierarchical clustering of the significance of overlap in binding between pairs of transcription factors. Ubx and Hth were compared to a set of embryo transcription factor binding profiles generated by MacArthur et al. Values represent −log10 transformed p-value for each pair-wise transcription factor comparison. C) Venn diagram comparing Ubx-bound regions in the embryo, haltere disc, or T3 leg disc. D) Multispecies conservation score for Ubx-bound regions in imaginal discs that overlap with Ubx-binding in the embryo (red, blue), and for regions that do not overlap with Ubx-binding in the embryo. Average score for a 1000 bp window (center of called peak plus/minus 500 bp) is represented.

When measured *in vitro*, homeodomain proteins such as Ubx and Hth have low DNA binding specificities as monomers. If these proteins bound to the majority of their six base pair binding sites, they would bind on average once per ∼1300 base pairs of genomic DNA. A simple inspection of our ChIP-chip binding data for a small region of the *Drosophila* genome indicates that this is not the case: only a tiny subset of Ubx monomer binding sites are actually bound *in vivo* (Figure 7A). Further, inspection of these binding patterns indicate that the pattern of binding in the T3 leg is distinct from the pattern of binding in the haltere, a conclustion that is reinforced by the individual gene snapshots shown in Figures 4 and 5.

To get an overall view of Ubx and Hth tissue specificity, we calculated genome-wide for each factor in leg versus haltere tissues (Supplementary Figures S3A and S3B). For both Ubx and Hth binding in these two tissues is positively correlated, though the correlation is stronger for Ubx (0.76) than for Hth (0.50). That is, on a broad scale Ubx is largely bound in a similar pattern in both haltere and leg discs, though the correlation is not perfect indicating there are tissue specific differences. This is also true, though to a lesser extent, for Hth, where tissue specific binding is more obvious. A positive correlation between Ubx and Hth binding profiles is also seen in each tissue (Supplementary Figures S3C and S3D). The correlation between Ubx and Hth is much stronger in the leg than in the haltere, which is perhaps not surprising considering that multiple Ubx functions in the haltere are known to be independent of Hth. We next explore the tissue specific binding of Ubx and/or Hth in more detail by focusing on peaks called as Ubx or Hth binding sites (see Methods for peak calling details).

A role for tissue-specific Ubx binding is supported by a global analysis of the binding sites. For this analysis, we included a comparison with the Ubx binding sites identified by ChIP-chip experiments using 0–12 hour embryos, previously performed by the modENCODE project (www.modencode.org; see also the individual gene snapshots in Figures 4 and 5, which include an embryo Ubx binding track). In contrast to individual imaginal disc types, such as the T3 leg or haltere, 0–12 hour embryos contain many cell types and tissues at multiple developmental stages, providing an interesting comparison with our data sets. Together, these comparisons reveal a remarkable amount of tissue-specific binding by Ubx. For example, of the 4590 Ubx binding sites identified in the haltere, only 16% were also identified in the T3 leg and approximately 42% identified in 0–12 hour embryos (Table 1 and Figure 7C). A significant amount of tissue specificity is also observed for Hth: of the 1062 Hth binding sites identified in the T3 leg, approximately 40% are also bound in the haltere (Table 1). Transcription factor binding specificities can also be seen by comparing the Hth and Ubx binding sites. This high degree of binding specificity is also observed when we compared the Ubx and Hth binding sites identified in the imaginal discs with the sites bound by a group of transcription factors analyzed by ChIP-chip in embryos [49], [50]. Specifically, the binding sites identified in embryos are more similar to each other than to those bound by either Ubx or Hth in the imaginal discs (Figure 7B). One interpretation of these data is that using a more purified population of cells, such as a single type of imaginal disc, reveals transcription factor binding specificities that are more likely to be blurred when the cell type diversity is greater as in embryos. Alternatively, there may be general differences in the chromatin architecture between the imaginal discs and embryos that result in these two data sets grouping independently. A third possibility is that the transcription factors in the embryo data sets have many more physiological target genes in common with each other than with Ubx and Hth. One argument against this third possibility is that the targets of Ubx in the embryo have limited overlap with the targets of Ubx in the T3 leg and haltere.

We also find that the imaginal disc-specific binding sites identified for Ubx (i.e. those sites identified in the haltere or T3 leg but not in the embryo) are more likely to be evolutionarily conserved in related species than the binding sites that are shared between imaginal discs and embryos (Figure 7D). Because evolutionarily conserved sites may be more functional than nonconserved sites [51], [52], this observation suggests that the tissue-specific binding sites are more likely to be functional than the binding sites that are shared in multiple tissues. It is also noteworthy that close to 50% of the Ubx sites that are shared between the embryo and haltere data sets are close to transcription start sites, and tend to be located upstream of housekeeping genes (Supplementary Figure S4B). A similar conclusion comes from a comparison between the embryo and leg Ubx bound sites: binding sites that are shared between two tissues have a stronger tendency to be located close to promoters. It is possible that some of this tissue-nonspecific promoter binding upstream of housekeeping genes is a result of open chromatin upstream of these highly transcribed genes and does not represent functional binding. These observations suggest that tissue-specific sites may be more functional than the non-tissue-specific sites.

Finally, we note that this overall picture does not change if we analyze sites bound by both Ubx and Hth: co-bound sites still show a similar degree of tissue specificity as sites that are bound by only Ubx or only Hth (e.g. only 22% of the Ubx+Hth sites identified in the haltere are also identified as Ubx+Hth sites in the T3 leg; Table 1).

### DNA motif analysis

**Figure 8:**
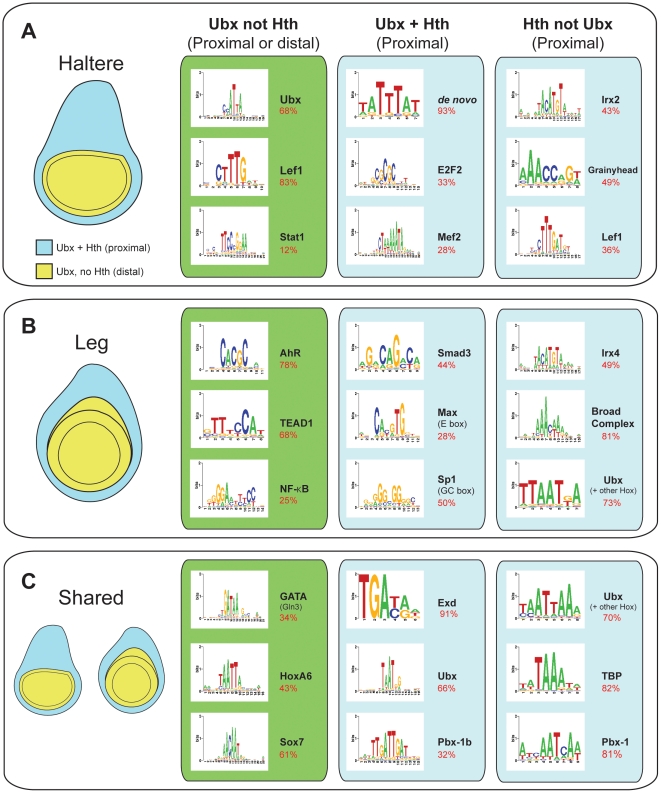
DNA motif analysis of Ubx, Hth, and Ubx+Hth bound fragments in the T3 leg and haltere. Select motifs identified using SeqPos/CEAS (Methods; all motifs p<0.01). The haltere and leg schematics on the left are divided into the proximal (blue, expressing Ubx and Hth) and distal (yellow, expressing Ubx but not Hth) regions to illustrate Ubx and Ubx+Hth expression patterns. Motifs in the left box were identified in regions bound by Ubx (but not Hth) in the indicated tissue(s); this box is green because Ubx (not Hth) binding could occur in either the proximal or distal regions of these discs. Motifs in the center box were identified in regions bound by Ubx+Hth in the indicated tissues(s), and motifs in the right box were identified in sequences bound by Hth (but not Ubx) in the in the indicated tissue(s). The top panel (A) represents sequences identified in the haltere-specific binding regions for Ubx and/or Hth. The middle panel (B) represents sequences identified in the leg-specific binding regions for Ubx and/or Hth. The bottom panel represents sequences identified in binding regions for the indicated factor(s) that are not tissue-specific (i.e., shared between the haltere and leg). For each motif, the percentage of peaks containing a matching sequence within +/− 250bp is given in red, below the motif name.

Because Ubx is important for generating both the T3 leg and haltere, two very distinct parts of the adult fly, it must be functioning with other selector-like genes to establish these fates. Moreover, the high degree of tissue specificity observed in our ChIP-chip data sets suggests that Ubx binding must be influenced by additional factors. As a first step towards identifying candidate factors that Ubx is collaborating with in either the T3 leg or haltere, we searched for the presence of statistically significant DNA sequence motifs in the bound DNA sequences. We searched for both known transcription factor binding sites using existing position weight matrices (PWMs) as well as *de novo* discovered motifs. We used these approaches to analyze nine groups of DNA sequences: 1) sequences bound by Ubx (but not Hth) in the haltere, T3 leg, or both discs (3769, 108, and 656 regions, respectively), 2) sequences bound by Ubx+Hth in the haltere, T3 leg, or both discs (89, 18, and 76 regions, respectively), and 3) sequences bound by Hth (but not Ubx) in the haltere, T3 leg, or both discs (80, 641, and 328 regions, respectively) (Figure 8). Because Hth is expressed only in a subset of these imaginal discs, these analyses have the ability to identify factors that may be collaborating with Ubx (and/or Hth) in different regions of these discs (Figure 8). In multiple cases, motif searches using these different sets of sequences identified distinct motifs, suggesting that Ubx and/or Hth are indeed working with different transcription factors in a tissue- and regional-specific manner.

Although the complete list of discovered motifs is provided in Supplementary Table S9, we highlight a few here (Figure 8). Although, and as discussed above (Figure 7A), only a tiny subset of Ubx monomer binding sites are actually bound by Ubx, motifs matching Ubx PWMs were identified multiple times in these data sets. Interestingly, motifs that match other *Drosophila* Hox PWMs (e.g. Abd-A and Scr), while prevalent in all of the Hth, not Ubx datasets, were not identified above a statistically significant threshold of p<0.01 in any of the Ubx, not Hth bound regions. It is also of interest that the Ubx monomer PWM was not identified in the leg-specific, Ubx not Hth set of bound regions, perhaps suggesting that, in the distal leg, Ubx cofactors other than Hth are shifting the specificity of Ubx binding away from the canonical TAAT motif. Also as expected, motifs matching the consensus binding site for Hox-Exd heterodimers [22], TGATnnAT, were discovered in two different data sets, consistent with a role for Ubx-Exd-Hth in the regulating a subset of target genes (labeled “Pbx-1b” in Figure 8).

A motif matching the PWM for Sp1, which is a selector gene for the legs in *Drosophila* [34], was discovered in the Ubx+Hth leg data set, but not in either of the Ubx haltere data sets. Also in the Ubx, not Hth leg data set, a motif matching the AhR motif was discovered. This is of interest because *spineless-aristapedia* (*ss*) is a member of this gene family and is expressed specifically in ventral, but not dorsal, imaginal discs, including the legs. In fact, *ss* is only expressed in the distal leg – the Ubx, not Hth domain – so this is a potentially interesting tissue- and region-specific cofactor of Ubx [53], [54].

A motif matching the PWM for Irx2, a member of the Iroquois gene family, was identified in the Hth, not Ubx haltere data set. This is of interest because the Iro genes are expressed and required in the Hth-expressing domains of the wing and haltere discs [55], [56]. Similarly, a motif matching the PWM for Grainyhead was identified in one of the haltere data sets (Hth, not Ubx), but was not discovered in any of the leg data sets. A motif matching the GATA PWM was discovered in both the leg and haltere, Ubx, not Hth, data sets. This motif is likely to bind *pannier* (*pan*), which encodes a GATA factor that is expressed in a subset of the wing and haltere imaginal discs [57].

Motifs matching PWMs for Lef1, which is a homolog of Pangolin (Pan), a transcription factor in the Wg signaling pathway [58], were identified in two haltere data sets (Ubx, not Hth and Hth, not Ubx). Finally, a motif matching the PWM for Smad, which the homolog of Mothers against Dpp (Mad), a transcription factor in the Dpp signaling pathway [59], [60], was identified in the Ubx+Hth leg data set. Consistent with a handful of examples described previously [13], [14], [15], [17], [61], [62], [63], these findings support the idea that Ubx collaborates, perhaps directly, with both the Wg and Dpp signaling pathways to regulate a large number of target genes in the haltere and T3 leg.

The general picture that emerges from these analyses is that the different data sets, which reflect tissue-specific binding of Ubx and/or Hth, identify distinct motifs. In several cases (e.g. motifs expected to bind Ss in the leg or motifs expected to bind Iro factors in the haltere), the proteins that are likely to bind these motifs are expressed in a tissue-specific manner. Thus, the motifs discovered by these analyses represent excellent candidates for binding factors that collaborate with Ubx and/or Hth in these tissues [22].

### Conclusions

In summary, we have used genome-wide ChIP-chip approaches to map the binding sites for two developmentally critical transcription factors in two tissues that give rise to very limited subsets of the adult fly. In particular, the haltere and T3 leg imaginal discs only give rise to the ectoderm of the dorsal and ventral portions, respectively, of the third thoracic segment. Both discs include cells that generate non-appendage portions of the body, as well as two very distinct appendages, the haltere and the T3 leg. Although the cells within these discs have distinct fates (for example, body versus appendage and differences along the proximodistal axis of the appendages), imaginal discs have no endodermal cells and no or very few mesodermal cells. Thus, compared to the fly embryo, which includes cells from all three germ layers, imaginal discs represent a much purer population of cells. Further, the imaginal discs used in these experiments came from a single time point in development – the end of the third instar larval stage – thus limiting another potential source of variability that is more problematic in ChIP experiments carried out with whole embyos.

Consistent with this limited amount of cell type diversity, and the fact that these tissues give rise to distinct parts of the adult fly, we found that the putative target genes regulated by these transcription factors are different in these two tissues. This makes sense, given that in the leg *Ubx* only needs to differentiate the T3 leg from the T2 leg, while in the haltere, *Ubx* must suppress wing fates and promote haltere fates. Although the most significantly enriched class of target genes encode transcription factors, we find that *Ubx* is bound to many genes that carry out a wide variety of functions. Many of these genes may represent the so-called ‘realizator’ genes conceived by Garcia-Bellido, which execute functions that are required for the terminal differentiation of specific cell fates [64], [65].

Tissue specificity is also reflected in the actual binding sites occupied by Ubx and/or Hth. Not only are the sets of ChIPed fragments different between the T3 leg and haltere, the associated DNA sequence motifs are also distinct.

Together, these data provide a rich source of information for understanding the complete network of genes required to make these two parts of the adult fly, as well as understanding how these transcription factors exhibit distinct DNA binding profiles in these two tissues. The suggestion from this work, which must be tested experimentally in the future, is that Ubx and/or Hth binding specificities are in part determined by the other regionally expressed transcription factors, some of which are likely to be represented by the motifs discovered here.

## Methods

### Chromatin Immunoprecipitation and ChIP-chip

Wandering third-instar larvae were dissected and imaginal discs were collected in PBS on ice. Discs were fixed with 1.8% formaldehyde, and crosslinked chromatin was sonicated to an average size of 500 bp. Chromatin preparation and immunoprecipitation were performed as described [66]. For imaginal disc ChIP, both goat anti-Hth (dG-20, Santa Cruz Biotechnologies) and rabbit anti-Ubx (Ubx1, generated by modENCODE, http://intermine.modencode.org/release-22/portal.do?class=Antibody&externalids=UBX1 ) were used at a final concentration of 1.5 µg/ml for each immunoprecipitation. Immunoprecipitated DNA and input DNA were amplified for array hybridization using the GenomePlex WGA4 Whole Genome Amplifcation Kit (Sigma). The samples were then labeled according to Affymetrix protocols and hybridized on Affymetrix GeneChip *Drosophila* Tiling 2.0R Arrays.

Binding data were processed with both MAT (Model-based Analysis of Tiling-arrays) [67] and TAS (Tiling Analysis Software, from Affymetrix). Peaks were called at 5% FDR (false discovery rate) using MAT, and a threshold of the top 5% of p-values was used to identify peaks with TAS. The intersection of these two peak-calling methods – peaks called using TAS (top 5%) whose center fell within 250bp of a 5% FDR peak called in MAT – was used to identify Ubx- or Hth-bound genomic regions. This dual threshold peak calling method was used to be more inclusive than 1% FDR (which identified very few peaks in a number of our datasets) and still limit the number of peaks called at 5% FDR. For example, at 5% FDR approximately approximately 11,000 regions are called as bound by Ubx in the haltere; filtering this set by those regions that also match the top 5% of p-values in TAS reduces this number to 4,590, which is a more manageable list of peaks that are identified by independent analysis methods.

Similar ChIP-chip data sets for Ubx and Hth binding in the haltere were generated using different reagents by Steven Russell, Robert White and Siew Woh Choo of the University of Cambridge (personal communication). Comparisons between our data and these data reveal a broadly similar datasets, providing additional validation for both data sets. Approximately 3000 (65%) of the Ubx haltere peaks identified in this study are also associated with Ubx in the ChIP experiments performed by Choo et al., and 432 (77%) of the Hth haltere peaks are also associated with Hth in the data generated by Choo et al. (Supplementary Figure S1B). We used both datasets to generate a set of high- and medium-confidence Ubx and Hth haltere binding sites. High confidence sites are those called in our study that overlap with the top 10% of peaks (TAS) from the Choo et al. ChIP-chip data; medium confidence sites overlap the top 20% of peaks from the Choo et al. data. These medium- and high-confidence peaks, and their associated target genes, can be found in Supplementary Tables S1, S2, S3, S4. GO analysis of the target genes for these high-confidence peaks revealed significant enrichment for developmental processes such as imaginal disc development (Supplementary Tables S5 and S6), again suggesting that these are biologically important binding events.

Embryo ChIP-chip was performed as described previously [68] as part of the modENCODE project. While multiple, largely consistent Ubx embryo datasets have been generated for modENCODE, the data analyzed here are from ChIPs performed with rabbit anti-Ubx (Ubx7701, http://intermine.modencode.org/release-22/portal.do?class=Antibody&externalids=KW0%3AUBX-7701) as this is the most inclusive of the Ubx embryo data. Peak calling for the embryo data was performed using the same criteria as was used for the imaginal disc data. Imaginal disc ChIP-chip data have been submitted to the Gene Expression Omnibus (GEO) under accession number GSE26793 and all data described here will be available through modENCODE.

### Drosophila strains, antibodies and immunohistochemistry

Wild-type flies used for ChIP were *yw*. The following *lacZ* reporters were used: *fj-lacZ* [69], *vn-lacZ* [70], *wg-lacZ* [71], *notum-lacZ* (notum^JW^), and *ex-lacZ* [72]. *ban-Gal4* is an enhancer trap line (P{GawB}NP0016) from the Drosophila Genetic Resource Center. The following antibodies were used for immunostaining: rabbit anti-βgal (Capell), guinea pig anti-Dll, mouse anti-ct (Developmental Studies Hybridoma Bank), and mouse anti-Bs/DSRF (Markus Affolter).

### Expression analysis

Gene expression microarray data are from Hersh et al. [12]. Briefly, wild-type haltere versus wild-type wing and *Cbx^1^* wing versus wild-type wing comparisons (MAS5 normalized data) were analyzed using Significance Analysis of Microarrays (SAM) [73]. Differentially expressed genes were called at FDR<1%. The list of 488 Ubx-responsive genes and the expression patterns of those genes bound by Ubx and/or Hth is provided in Supplementary Table S6.

### Computational analysis

Target genes were called as the transcription start site (TSS) nearest to the called Ubx or Hth binding region. For each peak the nearest by distance, on either strand, to the center of the peak call is identified. Transcripts associated with a given TSS were identified based on Flybase annotation, and the strand information of the TSS and the relative location of the peak call were used to determine if the distance measurement is upstream or downstream of the TSS. Strand information was not included in assigning target genes, so a peak could fall upstream or downstream of the TSS of its assigned target gene. The vast majority of peaks for both Ubx and Hth fall within 2kb of the assigned genes TSS (Supplementary Figure S4 and S5), making target gene assignment straightforward, though an obvious caveat with this approach is that we cannot guarantee that a binding site is regulating the nearest TSS. Source code for our target gene assignment program is available on request. Gene ontology analysis was performed using Functional Annotation Clustering as part of DAVID [74]. To avoid redundant GO categories, only the most significant (p<0.001, Bonferroni-corrected) GO biological process or molecular process category from each enriched functional cluster was selected for inclusion in Figures 2 and 3 and Supplementary Tables S7 and S8.

For Table 1 and Figures 7–8, regions were called as overlapping if the center-to-center distance of called peaks was less than 500bp. To explore the interaction between Hth and Ubx and a set of previously published transcription factors (Figure 7B) [49], a Fisher's exact test was employed to test whether each pair of factors overlapped significantly (based on the number of binding sites for each factor, the overlap for a given pair of factors, and the total number of binding sites for all factors). Hierarchical clustering was performed on the −log10 transformed p-value for each of these pair-wise comparisons.

Motif analysis was performed using SeqPos/CEAS, which is part of Cistrome (http://cistrome.dfci.harvard.edu/ap/), a Galaxy-based platform for ChIP-chip analysis [75], [76], [77]. Motifs from the TRANSFAC [78] and JASPAR [79] databases, and motifs generated by large-scale one-hybrid screening [20], [80] and protein-binding microarray studies [81] were scanned for enrichment in Ubx- and/or Hth-bound genomic regions. All called peaks were used for analysis with SeqPos (from as few as 18 for leg-specific Hth+Ubx to more than 3700 for haltere-specific Ubx, not Hth). As described previously, SeqPos scans for significant motif hits by testing relative entropy cutoffs greater than or equal to 3 for a given PWM; the cutoff resulting in the highest positional bias toward the center of a called peak is then used [76], [77]. Therefore, all motif hits as shown in Figure 8 scored above a relative entropy of 3. MDscan was also used for *de novo* motif discovery. Selected motifs are represented in Figure 8, and the entire SeqPos output, with significance values, can be found in Supplemental Table S9.

## Supporting Information

Figure S1 Comparison of Slattery et al. and Choo et al. Ubx haltere binding. A) Genome-wide correlation plot and correlation values (TAS log2 signal, 500bp sliding window) for haltere data generated in this study and the study by Choo et al. Ubx-Ubx comparison is on the left and Hth-Hth comparison on the right. B) Percent of haltere Ubx or Hth peaks called in this study that overlap peaks called from data generated by Choo et al. at various stringencies (top 5%, top 10%, and top 20% of TAS p-values). (C–E) Haltere disc Ubx and Hth binding profiles generated in this study and the study by Choo et al. at the following loci: BX-C HOX locus (C), ANT-C HOX locus (D), bantam (ban) (E), E2F (F), dally (G), frizzled 2 (fz2) (H).
(1.83 MB TIF)

Figure S2 Examples of Ubx- and Hth-bound genes. Ubx and Hth binding profiles in the T3 leg and haltere imaginal discs at the following genes: cyclin A (cycA) (A), Distalless (Dll) (B), dally-like protein (dlp) (C), four-jointed (fj) (D), frizzled (fz) (E), hedgehog (hh) (F), patched (ptc) (G), vein (vn) (H), spalt major (salm) (I), and knot (kn) (J). Enhancers near salm and kn previously shown to be targeted by Ubx are shown as yellow boxes along the chromosomal map. The salm enhancer is not called as targeted by Ubx using our dual threshold for calling peaks (Methods), however it is called as targeted by Ubx using the MAT 5% FDR threshold alone. Color scheme and tracks are as described in Figure 4.
(2.00 MB TIF)

Figure S3 Genome-wide Ubx and Hth correlations. Genome-wide correlation plots (TAS log2 signal, 500bp sliding window) for Ubx leg versus Ubx haltere (A), Hth leg versus Hth haltere (B), Hth haltere versus Ubx haltere (C), and Hth leg versus Ubx leg (D). Correlation values are indicated within each plot.
(1.24 MB TIF)

Figure S4 Binding site location analysis. A) Percent of Ubx- or Hth-bound regions (haltere dataset) mapping to the indicated genomic features. Promoter is defined as −1000bp to the transcription start site; intergenic regions are those that fall between a promoter and the next upstream gene. B) Comparison of Ubx binding events that are haltere-specific (relative to Ubx binding in the embryo) and Ubx binding events that are shared between the embryo and the haltere. As in (A), the percent of bound regions mapping to the indicated genomic feature is represented.
(2.27 MB TIF)

Figure S5 Distribution of Ubx and Hth binding around TSS. Histograms representing the location of binding sites around the transcription start sites of called target genes for Ubx in the haltere (A), Ubx in the leg (B), Hth in the haltere (C), Hth in the leg (D).
(1.40 MB TIF)

Table S1 High confidence Ubx haltere peaks and target genes (Slattery et al., Choo et al.) All Ubx haltere peaks called in this study that also overlap a top 10% Ubx peak (TAS p-value) called from the Choo et al. data. Called target gene(s) associated with each peak are also indicated, and enriched GO categories are represented in the second sheet.
(0.81 MB XLS)

Table S2 High confidence Hth haltere peaks and target genes (Slattery et al., Choo et al.) All Hth haltere peaks called in this study that also overlap a top 10% Hth peak (TAS p-value) called from the Choo et al. data. Called target gene(s) associated with each peak are also indicated, and enriched GO categories are represented in the second sheet.
(0.21 MB XLS)

Table S3 Medium confidence Ubx haltere peaks and target genes (Slattery et al., Choo et al.) All Ubx haltere peaks called in this study that also overlap a top 20% Ubx peak (TAS p-value) called from the Choo et al. data. Called target gene(s) associated with each peak are also indicated.
(0.36 MB XLS)

Table S4 Medium confidence Hth haltere peaks and target genes (Slattery et al., Choo et al.) All Hth haltere peaks called in this study that also overlap a top 20% Hth peak (TAS p-value) called from the Choo et al. data. Called target gene(s) associated with each peak are also indicated.
(0.07 MB XLS)

Table S5 Called Ubx and Hth target genes (this study). All genes called as Ubx or Hth targets in either the haltere or the leg. Whether Ubx or Hth targets a gene in a given tissue is indicated with a “yes” or “no” in each column.
(0.52 MB XLS)

Table S6 Ubx-responsive genes bound by Ubx or Hth. Ubx-responsive genes (see Methods) that are also bound by Ubx and/or Hth in the haltere and log2-transformed expression values in haltere or Cbx1 mutant wing (both relative to wild-type wing) are represented. All Ubx responsive genes are provided in second sheet.
(0.06 MB XLS)

Table S7 Significant Gene Ontology categories for haltere datasets. Biological process or molecular function GO categories with Bonferroni-corrected p-value<0.01 are represented for the indicated Ubx and/or Hth, haltere datasets.
(0.04 MB XLS)

Table S8 Significant Gene Ontology categories for leg datasets. Biological process or molecular function GO categories with Bonferroni-corrected p-value<0.01 are represented for the indicated Ubx and/or Hth, haltere datasets.
(0.03 MB XLS)

Table S9 Enriched DNA motifs. Top 100 significant DNA motifs (p<0.01) identified by SeqPos for binding categories described in Figure 8. Log-transformed p-values are also provided.
(0.74 MB DOC)
