# Supplementary figures and images for: Genome-Wide Tissue-Specific Occupancy of the Hox Protein Ultrabithorax and Hox Cofactor Homothorax in Drosophila
Source: PLoS One. 2011 Apr 5;6(4):e14686. doi: 10.1371/journal.pone.0014686 (PMC3071676; doi:10.1371/journal.pone.0014686)

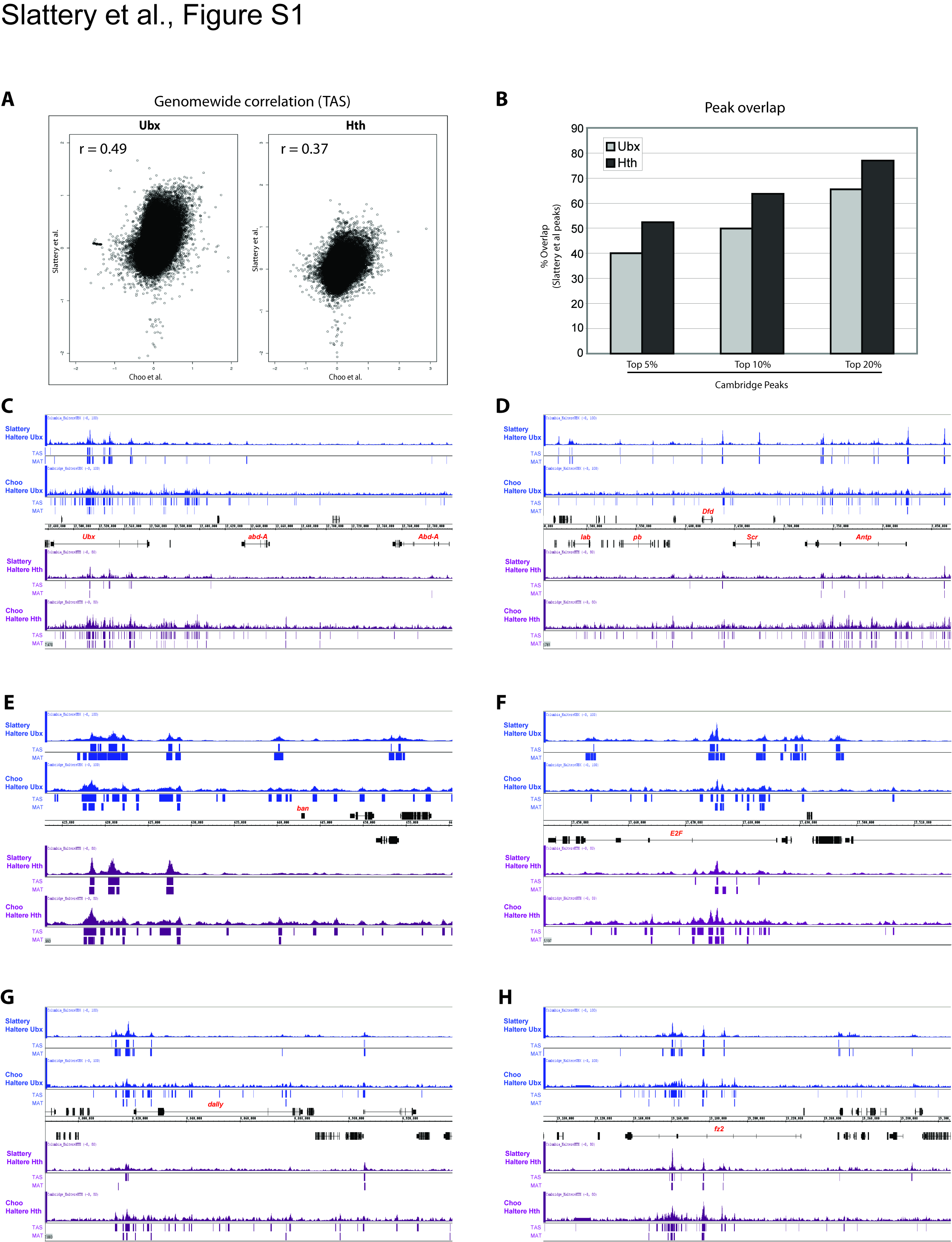

Supplement: Figure S1 — Comparison of Slattery et al. and Choo et al. Ubx haltere binding. A) Genome-wide correlation plot and correlation values (TAS log2 signal, 500bp sliding window) for haltere data generated in this study and the study by Choo et al. Ubx-Ubx comparison is on the left and Hth-Hth comparison on the right. B) Percent of haltere Ubx or Hth peaks called in this study that overlap peaks called from data generated by Choo et al. at various stringencies (top 5%, top 10%, and top 20% of TAS p-values). (C–E) Haltere disc Ubx and Hth binding profiles generated in this study and the study by Choo et al. at the following loci: BX-C HOX locus (C), ANT-C HOX locus (D), bantam (ban) (E), E2F (F), dally (G), frizzled 2 (fz2) (H). (1.83 MB TIF) [file pone.0014686.s001.tif]

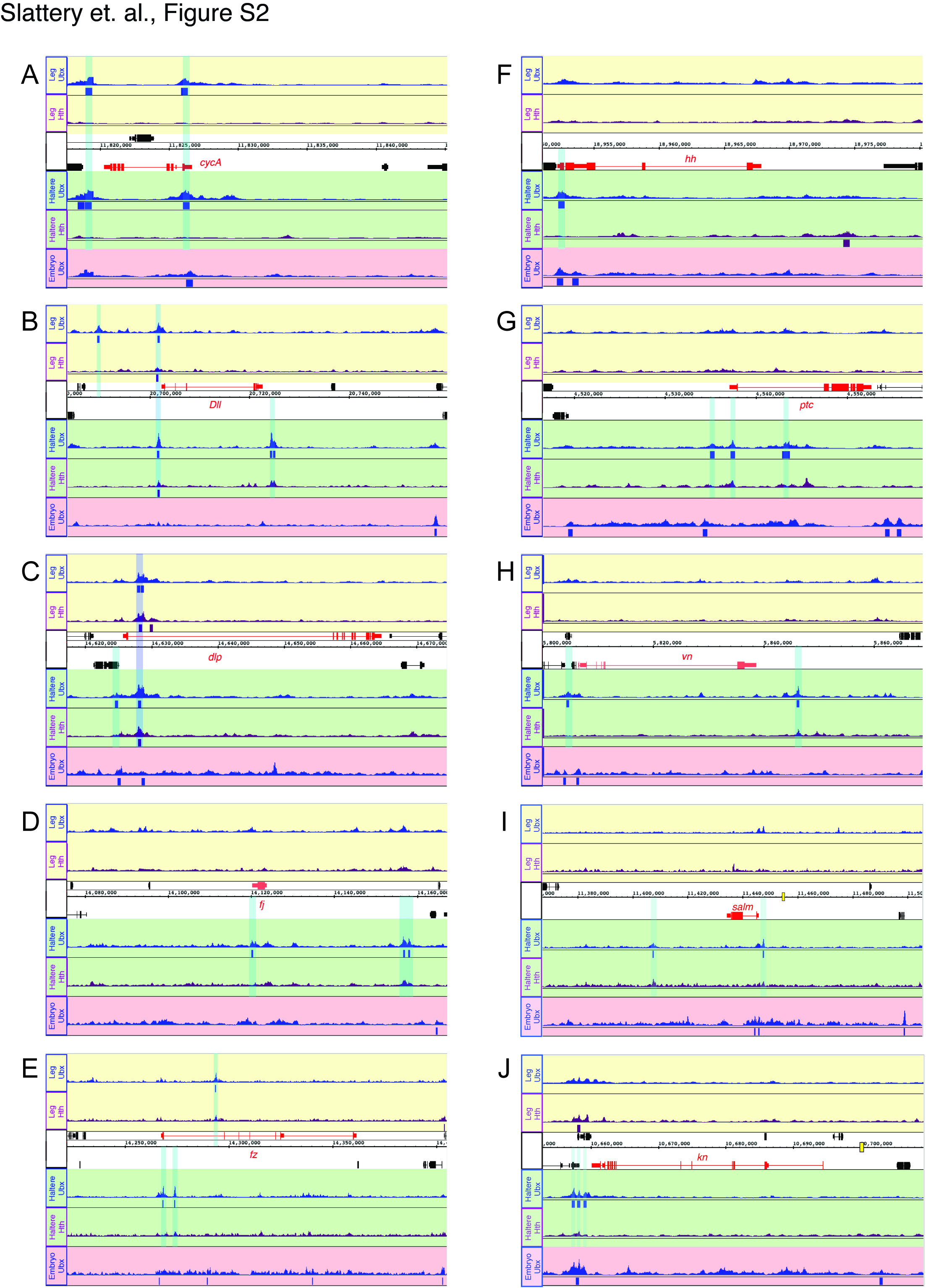

Supplement: Figure S2 — Examples of Ubx- and Hth-bound genes. Ubx and Hth binding profiles in the T3 leg and haltere imaginal discs at the following genes: cyclin A (cycA) (A), Distalless (Dll) (B), dally-like protein (dlp) (C), four-jointed (fj) (D), frizzled (fz) (E), hedgehog (hh) (F), patched (ptc) (G), vein (vn) (H), spalt major (salm) (I), and knot (kn) (J). Enhancers near salm and kn previously shown to be targeted by Ubx are shown as yellow boxes along the chromosomal map. The salm enhancer is not called as targeted by Ubx using our dual threshold for calling peaks (Methods), however it is called as targeted by Ubx using the MAT 5% FDR threshold alone. Color scheme and tracks are as described in Figure 4. (2.00 MB TIF) [file pone.0014686.s002.tif]

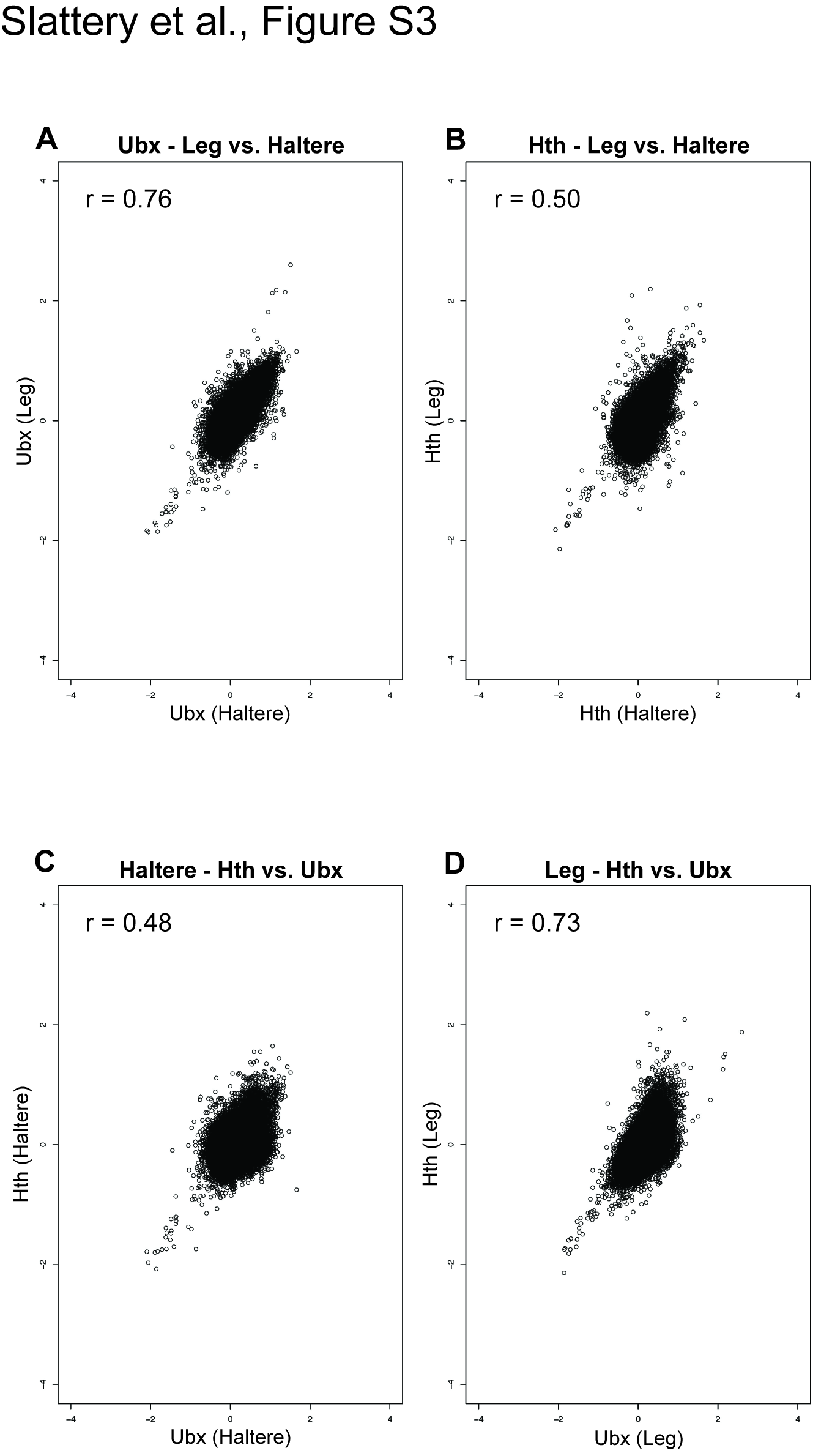

Supplement: Figure S3 — Genome-wide Ubx and Hth correlations. Genome-wide correlation plots (TAS log2 signal, 500bp sliding window) for Ubx leg versus Ubx haltere (A), Hth leg versus Hth haltere (B), Hth haltere versus Ubx haltere (C), and Hth leg versus Ubx leg (D). Correlation values are indicated within each plot. (1.24 MB TIF) [file pone.0014686.s003.tif]

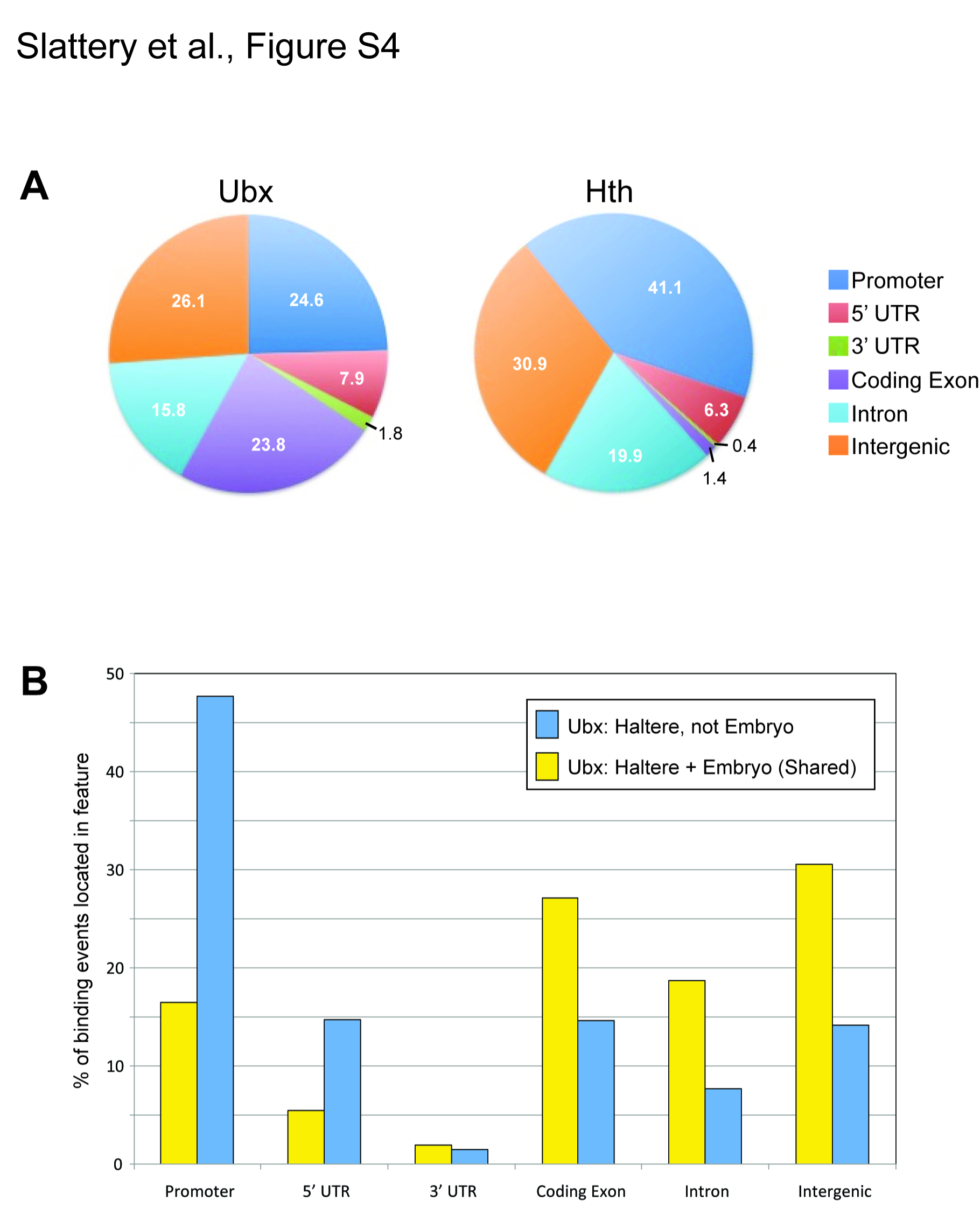

Supplement: Figure S4 — Binding site location analysis. A) Percent of Ubx- or Hth-bound regions (haltere dataset) mapping to the indicated genomic features. Promoter is defined as −1000bp to the transcription start site; intergenic regions are those that fall between a promoter and the next upstream gene. B) Comparison of Ubx binding events that are haltere-specific (relative to Ubx binding in the embryo) and Ubx binding events that are shared between the embryo and the haltere. As in (A), the percent of bound regions mapping to the indicated genomic feature is represented. (2.27 MB TIF) [file pone.0014686.s004.tif]

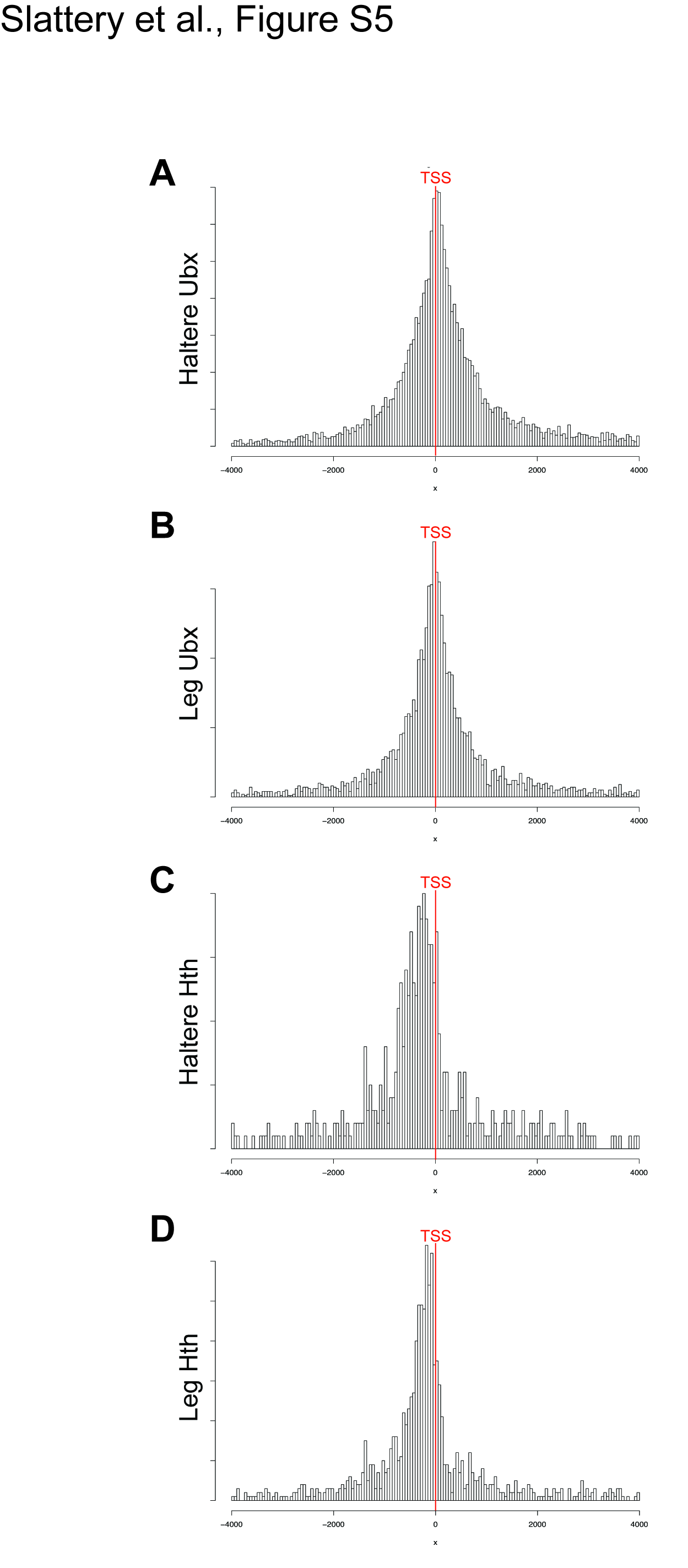

Supplement: Figure S5 — Distribution of Ubx and Hth binding around TSS. Histograms representing the location of binding sites around the transcription start sites of called target genes for Ubx in the haltere (A), Ubx in the leg (B), Hth in the haltere (C), Hth in the leg (D). (1.40 MB TIF) [file pone.0014686.s005.tif]
