# Supplementary material for: Genome-Wide Tissue-Specific Occupancy of the Hox Protein Ultrabithorax and Hox Cofactor Homothorax in Drosophila
Source: PLoS One. 2011 Apr 5;6(4):e14686. doi: 10.1371/journal.pone.0014686 (PMC3071676; doi:10.1371/journal.pone.0014686)
Supplement: Table S9 — Enriched DNA motifs. Top 100 significant DNA motifs (p<0.01) identified by SeqPos for binding categories described in Figure 8. Log-transformed p-values are also provided. (0.74 MB DOC) [file pone.0014686.s014.doc]

| **Haltere-specific Ubx, no Hth** |  |  |  |
| --- | --- | --- | --- |
| **Motif ID** | **factors** | **consensus** | **-10*LOG(pval)** |
| MA0068_observed | Pax4 | 1AAWA24C | 690.7755279 |
| M00380_observed | Pax-4 | 2AAA19CA4 | 690.7755279 |
| M00390_observed | FACB | 5KCC11GM5 | 690.7755279 |
| M00999_observed | AIRE | 6CC7CCA8 | 472.4331799 |
| M00955_observed | GR | 6AG1ACA15 | 387.1456965 |
| M00250_observed | Gfi-1 | 12GATTT7 | 315.8945462 |
| MB0184_observed | Gata3 | 7GAT4TC6 | 310.3046004 |
| M00957_observed | PR | 6A1AACA15 | 255.1234684 |
| M01087_observed | CEBP | 11K2CAMA5 | 236.699794 |
| MB0144_observed | Sox7 | 11WCAAT6 | 231.358109 |
| MB0072_observed | Aro80 | 8YCGG1W7 | 221.724574 |
| M00460_observed | STAT5A (homotetramer) | T1C3G1A8C6 | 199.8418972 |
| M00954_observed | PR | 6A1AACA15 | 191.680096 |
| M00956_observed | AR | 7GAACA15 | 190.474224 |
| M00138_observed | Oct1 | 8AT2AAA8 | 184.6653622 |
| MB0087_observed | Gln3 | 7GATAA9 | 171.4867553 |
| MB0024_observed | Skn7 | 8GGCCA8 | 166.7770894 |
| MB0060_observed | Rds2 | 6TCGG2T8 | 155.1821574 |
| M01036_observed | COUPTF | 5TG2CYY11 | 155.1105117 |
| MB0019_observed | Yap6 | 5T4TAA1C5 | 148.3441558 |
| M00015_observed | ABF1 | 5TC7ACG5 | 147.8322617 |
| MB0043_observed | Sfl1 | 6A2GAAG8 | 145.0630052 |
| M00388_observed | FACB | 5TS11SGA5 | 144.7028354 |
| M00327_observed | Pax-3 | 7GTS1CG8 | 143.7039257 |
| M01002_observed | DEAF1 | 6TCGG5T9 | 142.5214131 |
| MB0220_observed | Rfx3 | 7C1T1G2AC7 | 137.7321765 |
| M00143_observed | Pax-5 | 7C4C2S2TG8 | 127.4361877 |
| MB0074_observed | Oaf1 | 7CGG3TA6 | 123.5181544 |
| M01001_observed | DEAF1 | 8G6TCCG6 | 123.1633404 |
| M01075_observed | PLZF | 13TTTA10A1 | 122.4943954 |
| M00056_observed | myogenin / NF-1 | 13T1G6GCC4 | 120.2765871 |
| MB0005_observed | Ceh-22 | 7T2AGTG8 | 114.0049004 |
| MB0041_observed | Sip4 | 7TCCGG8 | 112.2421892 |
| MB0064_observed | Rdr1 | 8GCGGA8 | 110.9342597 |
| M00953_observed | AR | 6A1A1CA4G10 | 110.4605173 |
| M00023_observed | HOXA5 (Hox-1.3) | 13TAAT3G9 | 105.8680322 |
| MB0238_observed | Rfx3 | 11GCAAC7 | 102.9759738 |
| MB0207_observed | Gata3 | 8GATAA9 | 102.5984826 |
| M00057_observed | COMP1 | 11G1CAWT7 | 101.66464 |
| M00937_observed | TGA1a | 1A4T7RTY5 | 100.6297579 |
| MB0075_observed | Mcm1 | 5CC2W3RG5 | 95.84713873 |
| MB0038_observed | Yrm1 | 9T1TCCG6 | 93.03871136 |
| MB0023_observed | Aft1 | 9GCACC7 | 91.32268837 |
| M01070_observed | c-Maf | 5TGCTG9 | 90.62179021 |
| MB0071_observed | Ypr196w | 8TT2CCG6 | 90.57447626 |
| MB0003_observed | Oct1 | 7T1TK1A1A7 | 89.84480513 |
| M01011_observed | HNF1 | 7AA2A2AA5 | 89.07129498 |
| MA0066_observed | PPARG | 4GG6T2CC3 | 88.63069508 |
| MB0146_observed | Sox7 | 7AACAA10 | 88.55046381 |
| M00058_observed | HEN1 | 8CA1CTG8 | 82.94329679 |
| MB0143_observed | Bhlhb2 | 8CA1GTG8 | 82.0048644 |
| MB0086_observed | Gat1 | 7GATAA8 | 74.66581379 |
| MB0084_observed | Ynr063w | 7CGGAG8 | 73.11417014 |
| MA0007_observed | Ar | 4G1AC5G2C5 | 72.6958635 |
| MB0056_observed | Sut2 | 7CGGA1T7 | 69.81509468 |
| M00098_observed | Pax-2 | 4GTYA1G9 | 69.72144478 |
| M00259_observed | STAT | 6TT5AAA5 | 69.31597253 |
| M01051_observed | MCM1+SFF | 14AA6AA1A1 | 68.90111844 |
| MB0052_observed | Put3 | 8SGGRA7 | 68.88344885 |
| M00351_observed | GATA-3 | TWWDATCWTT | 67.2351889 |
| MB0092_observed | Gzf3 | 8GATAA7 | 67.22770605 |
| M00538_observed | HTF | 10ACGTG9 | 66.34451074 |
| M00038_observed | GCN4 | 10TG1C1CA10 | 65.35295572 |
| MB0012_observed | Pdr1 | 7T1CGGA7 | 62.29062055 |
| MB0011_observed | Cep3 | 7TCSG1A7 | 61.94070447 |
| M00373_observed | Pax-4 | 11C1YSA3C1 | 60.92700248 |
| MB0059_observed | Gsm1 | 6YCGG1G9 | 60.08000516 |
| M01022_observed | LEF1 | SWWCAAAGKS | 59.66732911 |
| M00068_observed | HEN1 | 8CA1CTG8 | 59.47218023 |
| MB0010_observed | Ypr015c | 7G1A1ATC6 | 59.3989729 |
| MB0051_observed | Mga1 | 9GAACA7 | 57.74226398 |
| 6_observed | RBM7 | AGGAG | 57.50811191 |
| M00224_observed | STAT1 | 6TT1C3AA6 | 56.62707712 |
| MB0025_observed | Nrg1 | 7G1CCCT7 | 56.43430831 |
| MB0017_observed | Ykl222c | 7CGGA1A8 | 56.06800965 |
| M00630_observed | FOXM1 | ARMWKSWBT | 56.04353516 |
| MB0299_observed | Bhlhb2 | 9CACG1G8 | 55.43808782 |
| M00261_observed | Olf-1 | 6TCCY4G7 | 55.0790324 |
| 21_observed | USP39 | TTTCMRR | 54.35764989 |
| MB0018_observed | Srd1 | 7GA1CT1C6 | 52.02480053 |
| MB0310_observed | Cutl1 | 6TRAT1A5 | 51.42117359 |
| MA0088_observed | znf143 | 6CCA3T1C5 | 50.6092383 |
| MB0085_observed | Stp4 | 9GGCTG7 | 50.28122305 |
| MA0036_observed | GATA2 | VGATR | 49.35036291 |
| MB0090_observed | Pho2 | 7WWWWW8 | 49.25582083 |
| M00097_observed | Pax-6 | 4TT3G2T3T4 | 49.05694366 |
| 123_observed | HIST2H2AB | CARAWRT | 49.03212407 |
| MB0057_observed | Rgt1 | 7T2TCCG6 | 48.93972886 |
| M01171_observed | BCL6 | KBTTCYAGGW | 48.71090767 |
| M00191_observed | ER | 11TGACC3 | 48.36245108 |
| M00018_observed | Ubx | 7TAAT1G6 | 48.28763839 |
| M00165_observed | HSF | 1GA5C2GA2 | 48.26378112 |
| 9_observed | CAT | SAGAART | 47.31674895 |
| M00229_observed | Skn-1 | YAWTGTCATYMW | 46.79915284 |
| MB0004_observed | Zif268 | 9CCC1C1C7 | 46.76258108 |
| M00197_observed | ABF1 | 2TC7ACG1 | 46.66503184 |
| M00284_observed | TCF11:MafG | 6TG5TCA6 | 46.16858226 |
| 150_observed | MYF6 | CARATG | 45.95913165 |
| MB0066_observed | Rap1 | 6G2CACC7 | 45.3985072 |
| MB0080_observed | Cbf1 | 8CAC1TG7 | 45.16701538 |

| **Leg-specific**  **Ubx, no Hth** |  |  |  |
| --- | --- | --- | --- |
| **Motif ID** | **factors** | **consensus** | **-10*LOG(pval)** |
| M00766_observed | LXR direct repeat 4 | GGG8G2C1 | 89.02194126 |
| M01057_observed | ERF2 | GGCGSCR | 81.29349092 |
| 120_observed | HHEX | WAATKRM | 75.44938092 |
| M00955_observed | GR | 15TGTT1T6 | 72.11485436 |
| M00999_observed | AIRE | 9GG1T5GG6 | 71.24643436 |
| 325_observed | HIST1H2BN | YSCMWGCRC | 68.96024356 |
| 175_observed | C9orf156 | KSCKGCG | 63.82021338 |
| M00413_observed | AREB6 | GNWMAGGTGWRT | 63.79187974 |
| M01023_observed | HSF1 | 1T1C2G5TC3 | 63.67701005 |
| MB0238_observed | Rfx3 | 7GTTGC11 | 63.26377999 |
| denovo12_observed |  | GRAKSGSARC | 63.138761 |
| MA0068_observed | Pax4 | 3G21TWTT1 | 61.88647997 |
| MA0119_observed | TLX1::NFIC | 2GC6CCA1 | 61.77215979 |
| MA0051_observed | IRF2 | 2AAA4AA7 | 57.04722917 |
| 311_observed | ZNF671 | CTGSCA | 56.29658788 |
| M00954_observed | PR | 15TGTT1T6 | 54.15682989 |
| M00957_observed | PR | 15TGTT1T6 | 52.63432965 |
| M00194_observed | NF-kappaB | 3GGA4CY2 | 51.26669235 |
| M01001_observed | DEAF1 | 6CGG1A5S8 | 50.91742318 |
| M00235_observed | AhR:Arnt | 3CACGC8 | 48.77832292 |
| M00634_observed | GCM | MMYRCCMGCAKD | 48.31343322 |
| 303_observed | RAB7A | CBGAGC | 47.1904462 |
| M00147_observed | HSF2 | MGAWBMTTCB | 46.77665613 |
| 25_observed | CPSF4 | GSAAAR | 46.12889907 |
| MB0187_observed | Irf3 | 3AA2G1AA3 | 45.83310852 |
| SW0013_observed | knirps.new.1 | BTGCYCYRSWTT | 45.25897005 |
| MA0451_observed | kni | BTGCYCYRSWTT | 45.24327798 |
| 42_observed | TCEAL2 | YCATTWM | 45.13138101 |
| denovo15_observed |  | GAGCAVRGMG | 44.84546744 |
| M00327_observed | Pax-3 | 7GTS1CG8 | 44.78537535 |
| M00725_observed | HP1 site factor | CTKKWRAAHWTT | 43.5223485 |
| 245_observed | ZNF323 | CGGACWTRA | 43.26478405 |
| MA0095_observed | YY1 | RATGGH | 42.81855561 |
| M00181_observed | E2 | 2A1C6GGT2 | 42.64208044 |
| MB0059_observed | Gsm1 | 6YCGG1G9 | 42.09216838 |
| MB0036_observed | Tec1 | 7CATTC8 | 41.52821492 |
| MB0263_observed | Osr2 | 7GTAGC4 | 41.08585485 |
| M00389_observed | FACB | 5GC6T6GC4 | 40.88521549 |
| MB0362_observed | Nkx2-6 | 5CACTT6 | 40.7731047 |
| 366_observed | TBPL1 | ATTAAWGSC | 40.52895564 |
| MB0267_observed | Irf4 | 5TCYCR5 | 40.33625827 |
| M00146_observed | HSF1 | MGMAYVTTCY | 40.00417156 |
| denovo7_observed |  | WTAAAAT | 39.99652755 |
| 264_observed | TEAD1 | STTKCCAT | 39.8771685 |
| M00056_observed | myogenin / NF-1 | 5G7CC7A2G3 | 39.66215235 |
| MB0184_observed | Gata3 | 7GAT4TC6 | 39.38418317 |
| MB0273_observed | Tcfap2a | 4CY3RGS3 | 39.06836479 |
| M00305_observed | HAP1 | 2CG2A1Y2C2 | 38.68676201 |
| MA0114_observed | HNF4A | 4CTTTG4 | 38.49799662 |
| 157_observed | NONO | CAAASCC | 37.76705708 |
| M01070_observed | c-Maf | 5TGCTG9 | 37.50925085 |
| MB0188_observed | Tcfap2c | 4CY3RGS3 | 37.23181086 |
| 91_observed | SMPX | GSYCYGG | 37.22684459 |
| MA0003_observed | TFAP2A | SCYBVSGGC | 36.43561979 |
| M00469_observed | AP-2alpha | SCYBVSGGC | 36.43561979 |
| M01091_observed | PRD | WAAYYRH | 36.14960071 |
| MB0245_observed | Mafk | 4AAA2GC4 | 36.13438086 |
| M00481_observed | AR | 1G2C5GT1C1 | 35.61964905 |
| M00374_observed | Opaque-2 | GATGAYRTGG | 35.17618845 |
| M00135_observed | Oct1 | 6TT2CA1A5 | 34.84111708 |
| MB0104_observed | Ascl2 | 6CAG1TG5 | 34.80857662 |
| 131_observed | IVD | GCTGATT | 34.70225968 |
| M00069_observed | YY1 | 10ATGGY5 | 34.4505523 |
| 290_observed | XRCC1 | AGCAMWTT | 34.01867606 |
| 49_observed | ZDHHC15 | CCATKSRDA | 33.83986345 |
| MB0097_observed | Rfx4 | 3GT1GC1A5 | 33.77607816 |
| MB0163_observed | Tcfap2e | 2TTT1TT6 | 33.54838743 |
| M00964_observed | PXR, CAR, LXR, FXR | RRRGTYMRKRRM | 33.54237463 |
| MB0393_observed | Hoxc9 | 4TT1ATK6 | 33.23625416 |
| MB0119_observed | Tcfap2b | 2SCC4GG3 | 32.88252699 |
| 367_observed | VPS4B | KYGCCC | 32.79900527 |
| 197_observed | PPP5C | RTGGM | 32.5904699 |
| M00247_observed | PacC | 7C1TGGC4 | 32.42579552 |
| 110_observed | GTF2B | RTTGCS | 32.37625507 |
| MA0222_observed | exd | WTTTGACR | 32.35742267 |
| M00031_observed | MATalpha2 | WWWDACAYGM | 32.19601088 |
| M00511_observed | ERR alpha | 6AGG1CA2 | 32.06231107 |
| 3_observed | FAM127B | GTKSCCM | 31.62897273 |
| denovo13_observed |  | YRGCGAKYKC | 31.4990962 |
| M00445_observed | Xvent-1 | 3MAAAY5 | 31.22679674 |
| 258_observed | SOD1 | GGCTC | 30.88242377 |
| denovo0_observed |  | GATGGTS | 30.83733231 |
| 322_observed | GTPBP6 | CATTHRA | 30.70843826 |
| M00226_observed | P | SKGGTWGGT | 30.66970521 |
| MB0179_observed | Sox1 | 6T1AATT4 | 30.53912386 |
| MB0484_observed | Cgd2_3490 | 4GCA1GC4 | 30.21953053 |
| MA0252_observed | vis | CTGTCA | 30.20270898 |
| SW0116_observed | Vis | CTGTCA | 30.20270898 |
| MB0461_observed | Pou3f3 | 6TGCA1A5 | 30.19492321 |
| M00683_observed | XBP1 | CTCGAWG | 30.13712962 |
| MB0446_observed | Nkx2-5 | 5CACTT6 | 29.91222458 |
| MA0258_observed | ESR2 | 2G7TG1CC3 | 29.75282797 |
| MB0157_observed | Sox13 | 5GG2GGG5 | 29.64582505 |
| M00328_observed | Pax-8 | 5CA1GCR7 | 29.63439348 |
| MB0072_observed | Aro80 | 7W1CCGR8 | 29.61137629 |
| M01014_observed | SOX | 6CAAAG2 | 29.1185079 |
| MB0308_observed | Dobox4 | 7ATMCC5 | 29.09444492 |
| M00952_observed | PCF5 | GYGGYCCCRM | 29.03426603 |
| 185_observed | POLE3 | ATGRMTG | 28.92724523 |
| M00778_observed | AhR | KSRCACGCVMR | 28.84262458 |

| **Shared**  **(Haltere and Leg)**  **Ubx, no Hth** |  |  |  |
| --- | --- | --- | --- |
| **Motif ID** | **factors** | **consensus** | **-10*LOG(pval)** |
| M00380_observed | Pax-4 | 2AAA19CA4 | 254.2180209 |
| MA0068_observed | Pax4 | 1AAWA4M20 | 218.7246641 |
| M01036_observed | COUPTF | 11RRG2CA5 | 120.6332386 |
| 374_observed | RBM8A | TGTGTM | 106.1382158 |
| MB0146_observed | Sox7 | 7AACAA10 | 102.5608511 |
| MB0203_observed | Mafb | 5GCA1AA4 | 102.5381128 |
| M00390_observed | FACB | 5KCC11GM5 | 76.72914169 |
| M00419_observed | MEIS1 | YWRTGACAGVDS | 73.04211093 |
| MB0144_observed | Sox7 | 11WCAAT6 | 70.19413361 |
| 274_observed | TP73 | GCSVAA | 70.05246836 |
| MB0024_observed | Skn7 | 9GGCCA7 | 69.63151717 |
| M00999_observed | AIRE | 8TGG7GG6 | 64.3775165 |
| M00143_observed | Pax-5 | 8CA2S2G5M6 | 63.11724909 |
| 293_observed | ZNF26 | SCCAAA | 62.78560125 |
| M00957_observed | PR | 15TGTT1T6 | 62.19871928 |
| M00528_observed | PPAR | 5GG2AA1G4 | 61.60309325 |
| MB0020_observed | Nhp6a | 8TA1ATW7 | 61.53607077 |
| 172_observed | RNF138 | ATGAA | 59.58300601 |
| MB0074_observed | Oaf1 | 7CGG3TA6 | 59.52859408 |
| MB0407_observed | Six1 | 5TGAT2C5 | 59.03893327 |
| MB0431_observed | Hoxc8 | 5AATTA6 | 56.87308912 |
| MB0332_observed | Hoxa6 | 4TAATT7 | 56.79958532 |
| MB0039_observed | Ndt80 | 8C1CAAA7 | 56.32783617 |
| M00388_observed | FACB | 5TS11SGA5 | 55.7130789 |
| MB0477_observed | Cdx2 | 7ATAAA4 | 55.32193304 |
| MB0416_observed | Six6 | 5TGATA7 | 55.29543494 |
| M00819_observed | Knox3 | BVBSTGACVGDK | 52.29724575 |
| 15_observed | ZNF238 | ACAKCTGK | 51.97350489 |
| SW0111_observed | Six4 | TGABAM | 51.77286278 |
| MA0204_observed | Six4 | TGABAM | 51.77286278 |
| MA0012_observed | br | WAAACWARWWS | 51.72954244 |
| 279_observed | UBB | YGGGM | 51.59634267 |
| M00953_observed | AR | 15TGTTC7 | 51.40168974 |
| MB0082_observed | Ydr520c | 6CGGAG9 | 51.1619583 |
| denovo13_observed |  | AAMAMMAAYA | 50.74374135 |
| MA0087_observed | Sox5 | WAACAAT | 50.51941781 |
| SW0112_observed | So | TGATAY | 49.32591506 |
| MA0246_observed | so | TGATAY | 49.32591506 |
| M00821_observed | Nrf-2 | 2TGA4GS2 | 49.01637402 |
| M00038_observed | GCN4 | 10TG1C1CA10 | 48.44341498 |
| M00117_observed | C/EBPbeta | 3TTGC3A3 | 48.34608508 |
| 241_observed | RXRA | TGACCY | 47.07236135 |
| M00391_observed | UAY | 2CGG7CG2 | 46.94176278 |
| 268_observed | NR2F1 | YGACCY | 46.4633599 |
| 366_observed | TBPL1 | GSCWTTAAT | 46.11792062 |
| M00116_observed | C/EBPalpha | 3TTR3MA3 | 46.07793624 |
| 43_observed | ZBTB46 | AGCAATT | 45.97797432 |
| M00958_observed | ABI4 | VRSCRCCGCCS | 45.72219086 |
| M00729_observed | Cdx-2 | 4ATWAA5 | 45.00449875 |
| MB0141_observed | Sox30 | 5ACAAT6 | 44.93181684 |
| MB0264_observed | Foxj1 | 5A1AACA4 | 44.8215641 |
| 103_observed | SND1 | CCSAAACY | 44.68369144 |
| M00940_observed | E2F-1 | SSCGSSAAAH | 44.60550496 |
| 353_observed | CCDC16 | ARRTGAA | 44.13145854 |
| 209_observed | BARX1 | AATGVAA | 43.93532575 |
| M00325_observed | NRSE | 4G3C2GG3G4 | 43.33084101 |
| M00723_observed | GAGA factor | RSWGAGMRMRR | 43.27235465 |
| M00073_observed | deltaEF1 | HYYCACCTKRR | 42.94455414 |
| M01087_observed | CEBP | 5R8CAMA5 | 42.90578448 |
| MB0169_observed | Sox13 | 6TTGTT5 | 42.26117502 |
| 85_observed | SF3B1 | GMCAGAY | 41.48568462 |
| MA0116_observed | Zfp423 | K3CC3GG4 | 41.37043317 |
| MB0079_observed | Cha4 | 7GCGGA9 | 41.33293312 |
| MB0087_observed | Gln3 | 7GATAA9 | 41.18432358 |
| M00538_observed | HTF | 10ACGTG9 | 41.11388997 |
| 108_observed | ZNF326 | GCMMAAY | 40.99437509 |
| MB0369_observed | Gsc | 5GGATT7 | 40.34925373 |
| MB0043_observed | Sfl1 | 6A2GAAG8 | 40.12893616 |
| 304_observed | DUSP26 | GSSAAAGS | 39.15177977 |
| M01011_observed | HNF1 | 7AA2A2AA5 | 39.12673217 |
| M00517_observed | AP-1 | 3TGA2MA3 | 38.65616633 |
| MB0341_observed | Six4 | 6GA1ACC5 | 37.93173872 |
| M01091_observed | PRD | DYRRTTW | 37.88081196 |
| MB0067_observed | Lys14 | 6CGG1A1T7 | 37.66797753 |
| M00473_observed | FOXO1 | MRWAAACAAA | 37.45026615 |
| MB0212_observed | Sox8 | 6ACAAT6 | 36.72769912 |
| M00005_observed | AP-4 | 5CAGC4G4 | 36.36097269 |
| 373_observed | GIT2 | TTGSAW | 36.24665986 |
| MB0017_observed | Ykl222c | 7CGGA1A8 | 35.95061296 |
| 254_observed | BOLL | MAMMRCA | 35.4731045 |
| 72_observed | FLJ37078 | TYKGAAR | 35.42046915 |
| MB0285_observed | Smad3 | 6CAGAC6 | 35.31909894 |
| 56_observed | DDX53 | TGTGT | 35.14522867 |
| M00099_observed | S8 | 7AATTA4 | 35.07691873 |
| 245_observed | ZNF323 | CKGACWTRA | 34.57228196 |
| M00389_observed | FACB | 4GC12TGC5 | 34.54724749 |
| M00809_observed | FOX factors | 2A2AAA1A3 | 34.37560581 |
| MB0220_observed | Rfx3 | 7C1T1G2AC7 | 34.32688049 |
| M00792_observed | SMAD | AGACWSCMY | 34.30337871 |
| M00456_observed | FAC1 | 4TKTT1T4 | 34.19952158 |
| MB0127_observed | Zfp128 | 5G1C1TAC5 | 34.12612284 |
| MB0311_observed | Dlx4 | 6AATTA6 | 34.02348043 |
| M01002_observed | DEAF1 | 6TCGG5T9 | 33.99438929 |
| MA0051_observed | IRF2 | 2A1A3AAA7 | 33.50181126 |
| M01162_observed | OG-2 | CAATTA | 33.46766016 |
| MB0174_observed | Bbx | 5GT1AAC6 | 33.37743824 |
| MB0007_observed | Matalpha2 | 7ACAAT8 | 33.2905348 |
| M00058_observed | HEN1 | 8CA1CTG8 | 33.18143827 |
| MA0126_observed | ovo | DCWGTTWCW | 33.16543782 |
| MA0239_observed | prd | DCWGTTWCW | 33.16543782 |

| **Haltere-specific Ubx+Hth** |  |  |  |
| --- | --- | --- | --- |
| **Motif ID** | **factors** | **consensus** | **-10*LOG(pval)** |
| M01083_observed | Abd-A | WWWWATTTVM | 159.4894377 |
| denovo18_observed |  | ATAAAWTAMA | 105.9367911 |
| denovo0_observed |  | AATWWAT | 88.40226141 |
| denovo5_observed |  | TWWTTTA | 70.1300469 |
| denovo6_observed |  | TCTTTTG | 68.67381385 |
| MB0022_observed | Sfp1 | 7AAA2TT7 | 66.36812531 |
| MB0098_observed | Zfp161 | 2CGCGC7 | 60.21016949 |
| M00232_observed | MEF-2 | 6T1TTT3G7 | 56.60062284 |
| 34_observed | SFT2D1 | WGSMAAW | 54.72432687 |
| MB0281_observed | Sox4 | 6ACAAT6 | 54.22838049 |
| M01091_observed | PRD | DYRRTTT | 53.85532401 |
| 21_observed | USP39 | TTTCMRR | 53.32039624 |
| MB0026_observed | Rpn4 | 7GCC1CC7 | 52.03716529 |
| M00651_observed | NF-muE1 | CSGCCATYK | 51.91078616 |
| MB0042_observed | Nhp6b | 7WWATA8 | 50.41568841 |
| 175_observed | C9orf156 | CSCAGCC | 49.14838869 |
| M00283_observed | Zeste | 5CACTC6 | 49.11274239 |
| MB0211_observed | E2F2 | 4SSCGC6 | 48.9178616 |
| MB0043_observed | Sfl1 | 5T3TTC2T6 | 48.16533399 |
| MB0318_observed | Hoxd10 | 6TTTAT6 | 47.56423401 |
| M00423_observed | FOXJ2 | 5AA1R1TT2 | 47.40772292 |
| MB0160_observed | Zfp410 | 5C2CCCC5 | 47.29724847 |
| 82_observed | FLI1 | CGGAART | 47.02209861 |
| SW0037_observed | H20 | TTWATDR | 46.92472514 |
| MA0448_observed | H2.0 | TTWATDR | 46.92472514 |
| MA0264_observed | ceh-22 | VSCACTYSAMM | 46.2818296 |
| MB0144_observed | Sox7 | 11WCAAT6 | 46.13051159 |
| MB0142_observed | Sox15 | 3AAT2CA5 | 46.12496961 |
| M00104_observed | CDP CR1 | SCVATCRATT | 45.28579584 |
| 152_observed | NAP1L1 | SCCTGR | 44.31216879 |
| 168_observed | KLF3 | WSCMATT | 44.12485842 |
| 105_observed | AFF4 | CCCWSC | 43.19314781 |
| 46_observed | CSTF2 | TTYATTT | 42.8686192 |
| MB0005_observed | Ceh-22 | 8CACT2A7 | 42.77090209 |
| MB0388_observed | Nkx2-2 | 5CACT2A5 | 42.50366993 |
| M00172_observed | AP-1 | WYKKWGTCASY | 42.42056018 |
| M00616_observed | AFP1 | AWWAAYWRCAY | 42.38030192 |
| M00026_observed | RSRFC4 | 4T2TT1TA4 | 41.49772709 |
| SW0060_observed | CG340131 | TTWATTG | 41.32732023 |
| MA0444_observed | CG34031 | TTWATTG | 41.32732023 |
| 127_observed | HSF1 | GMAMTTK | 41.1751084 |
| denovo7_observed |  | TTTRTTG | 41.02638865 |
| 329_observed | TAGLN2 | GSYCMGG | 40.68209089 |
| M00101_observed | CdxA | WWWWMTR | 40.6331112 |
| MB0097_observed | Rfx4 | 5T2CAAC3 | 40.6058102 |
| 106_observed | GPD1 | SRGSWGGG | 39.92961948 |
| M00390_observed | FACB | 5KC3T8G4W1 | 39.29932426 |
| M00284_observed | TCF11:MafG | 7GC4TCA6 | 39.26172641 |
| 288_observed | VIL2 | CGSARG | 39.07085216 |
| M00231_observed | MEF-2 | 6T1TTT1T9 | 38.47546986 |
| 63_observed | EEF1D | TGMCARKMA | 38.08609384 |
| 110_observed | GTF2B | SGCAAY | 38.06492504 |
| M00926_observed | AP-1 | TDWGTCAB | 37.86712814 |
| denovo1_observed |  | WATTTAT | 36.96911626 |
| M00967_observed | HNF4, COUP | ARRGKCMAR | 36.93490067 |
| SW0089_observed | Unc4 | TTAATTG | 36.90080175 |
| MA0250_observed | unc-4 | TTAATTG | 36.90080175 |
| denovo29_observed |  | TK1T2K4T1 | 36.44479867 |
| 166_observed | MRPL2 | TYYCYTGHY | 35.92914943 |
| MA0068_observed | Pax4 | 1AAWA24C | 35.83618943 |
| M00478_observed | Cdc5 | GAKTTAAMRWAW | 35.59782881 |
| 370_observed | GTPBP1 | TCAYAA | 35.46859194 |
| 95_observed | PRDX5 | CGTCST | 35.43049006 |
| MB0345_observed | Hoxb8 | 4TT1AT1G5 | 35.32388657 |
| M00518_observed | PPARalpha:RXRalpha | 5CC3A3CC3 | 35.18799184 |
| M00133_observed | Tst-1 | 2A1T2WA1T4 | 34.78975156 |
| M00020_observed | Ftz | CTTAATTRYWWT | 34.50146058 |
| 323_observed | ZRSR2 | MAKTT | 34.4989405 |
| MB0328_observed | Hmx1 | 5TT1A1TG5 | 34.42519501 |
| MB0055_observed | Mig2 | 8CCCC2A6 | 33.31624678 |
| M00259_observed | STAT | 6TT5AAA5 | 32.86458976 |
| 359_observed | KLF4 | TSAGAAA | 32.74177141 |
| M00257_observed | RREB-1 | 1C1CAMM7 | 32.70590261 |
| M00934_observed | Zeste | YCRCTCRAMWW | 32.64841232 |
| M01175_observed | CKROX | KGGSMGGGV | 32.44398782 |
| MB0024_observed | Skn7 | 8GGCCA8 | 32.32898687 |
| 91_observed | SMPX | GSYCYGG | 32.2295915 |
| SW0013_observed | knirps.new.1 | AAWVTRGRGCAV | 32.19025836 |
| MA0451_observed | kni | AAWVTRGRGCAV | 32.19025836 |
| M00116_observed | C/EBPalpha | 3T3SCAA3 | 32.17177268 |
| MA0182_observed | CG4328 | YMATWWW | 32.16204396 |
| SW0036_observed | CG4328 | YMATWWW | 32.16204396 |
| MB0474_observed | Pitx3 | 7AATCC4 | 31.91576856 |
| MA0088_observed | znf143 | 6CCM3T1C5 | 31.72278539 |
| 303_observed | RAB7A | CBGAGC | 31.68420381 |
| M01123_observed | Nanog | RGRVCCATTKCC | 31.5049314 |
| MA0087_observed | Sox5 | WAACAAT | 31.32698129 |
| 273_observed | TMSL3 | CGYCST | 31.31116811 |
| 227_observed | RAB2A | GACGMT | 31.22248331 |
| 199_observed | DDEFL1 | GYMAYYTACT | 31.16228988 |
| 74_observed | R3HDM2 | AWTTTA | 31.08632251 |
| SW0061_observed | Hmx | TTAATTG | 30.99760344 |
| MA0192_observed | Hmx | TTAATTG | 30.99760344 |
| 72_observed | FLJ37078 | TTTCMRA | 30.97167171 |
| M00751_observed | AML1 | ACCACA | 30.71836082 |
| M00388_observed | FACB | 5TC3W8SA5 | 30.6626204 |
| denovo9_observed |  | AATAAMT | 30.31483811 |
| MA0244_observed | slbo | ATKGCMMA | 30.3117294 |
| M00622_observed | C/EBPgamma | 1Y1A1K2A1A2 | 30.0241455 |
| denovo14_observed |  | YTGYWKYTGY | 29.75478768 |

| **Leg-specific Ubx+Hth** |  |  |  |
| --- | --- | --- | --- |
| **Motif ID** | **factors** | **consensus** | **-10*LOG(pval)** |
| M00792_observed | SMAD | RKKSDGKCT | 59.87034375 |
| MB0388_observed | Nkx2-2 | 5CACT2A5 | 58.27069379 |
| M00629_observed | Eve | KCWSSBCHKC | 48.78686403 |
| M00712_observed | myogenin | CASCTGYY | 48.05794216 |
| MB0104_observed | Ascl2 | 5CAG1TG6 | 44.16286871 |
| M00941_observed | MEF-2 | DKYTWTWTTWAR | 42.08073328 |
| M01099_observed | KNI | 5RAA2RG6 | 40.66223687 |
| 155_observed | NFIL3 | TKCAAT | 38.55831726 |
| M01173_observed | SREBP1 | 4SR1GTG5 | 37.15593108 |
| M00701_observed | SMAD3 | AGRCAGMCR | 35.40218099 |
| M00222_observed | Hand1:E47 | 4CCAGA7 | 34.00427678 |
| 242_observed | PKNOX2 | GCTGYC | 33.44722535 |
| denovo20_observed |  | 1S1G2G2G2G | 33.29835319 |
| 369_observed | H2AFY | VGATKGCBG | 33.28941838 |
| MA0264_observed | ceh-22 | DSCACTYSAVW | 32.29557673 |
| M00390_observed | FACB | 5KC8A2KG6 | 31.4965298 |
| M00961_observed | VDR | DGGKTCAYCSRG | 31.15101567 |
| MB0005_observed | Ceh-22 | 8CACT2A7 | 30.71426118 |
| 289_observed | WHSC2 | KCCAAAK | 29.98175255 |
| 169_observed | ECSIT | CWWTTC | 29.67883671 |
| denovo9_observed |  | TTGYTGT | 29.35672517 |
| MB0024_observed | Skn7 | 8SGCCM8 | 28.9196703 |
| MB0225_observed | Zbtb3 | 5C2TGCA5 | 28.31337105 |
| 73_observed | FHL2 | TSCCYAGY | 28.1631493 |
| 43_observed | ZBTB46 | AGCMWTT | 28.08788084 |
| M00123_observed | c-Myc:Max | DSKCAYRTGSTK | 27.47576161 |
| 290_observed | XRCC1 | MGCAMWTT | 27.43185272 |
| denovo7_observed |  | AWAYACA | 27.12525199 |
| 128_observed | HSPA1L | TGSCAG | 26.7809342 |
| MA0080_observed | SPI1 | AGRAART | 26.59445768 |
| 138_observed | PHOX2A | AATTAS | 26.40340152 |
| M01043_observed | Nkx2-5 | HSCCACTTSM | 26.35976082 |
| 146_observed | MEIS3 | GAYRGCTS | 26.18858702 |
| M00194_observed | NF-kappaB | 2KGGA5C2 | 25.67879647 |
| denovo3_observed |  | GGAGGAG | 25.15852574 |
| 6_observed | RBM7 | AGGAG | 25.00097448 |
| 258_observed | SOD1 | GGCTC | 24.56552456 |
| MB0079_observed | Cha4 | 7GCGGA9 | 24.51932328 |
| 292_observed | ZNF3 | BCATTTYR | 24.22258665 |
| MB0305_observed | Rhox11 | 5GCTGT7 | 23.93177419 |
| 315_observed | ESX1 | GMAGC | 23.33735261 |
| MB0440_observed | Rhox11 | 5GCTGT7 | 22.96603021 |
| MB0118_observed | Ascl2 | 4CCC3CC4 | 22.37640404 |
| M00344_observed | RAV1 | RBVBCAGGTGAW | 22.34739546 |
| 222_observed | ZBTB4 | HGCAAADS | 22.33805578 |
| M00196_observed | Sp1 | 3RGG1GG4 | 21.15441846 |
| M00726_observed | USF2 | CAKGBG | 21.14032986 |
| MB0043_observed | Sfl1 | 9GAAGR7 | 20.83449563 |
| M00277_observed | Lmo2 complex | SYSCAKSTGSAS | 20.78402082 |
| M00973_observed | E2A | RRCAGGTG | 20.76087174 |
| M00448_observed | Zic1 | GRCCMCCCM | 20.05893726 |

| **Shared**  **(Haltere and Leg), Ubx+Hth** |  |  |  |
| --- | --- | --- | --- |
| **Motif ID** | **factors** | **consensus** | **-10*LOG(pval)** |
| MB0176_observed | Glis2 | 2TA5AAA2 | 70.73963358 |
| 216_observed | ZNF695 | ARGGTYW | 64.40066828 |
| denovo25_observed |  | 3A1A1A1A1A1 | 62.74623531 |
| MA0036_observed | GATA2 | YATCB | 62.02679528 |
| denovo13_observed |  | ATWWTWWKTA | 61.75916371 |
| M01091_observed | PRD | DYRRTTW | 61.39593395 |
| M00421_observed | MEIS1B:HOXA9 | T1ACA2T6 | 58.07078456 |
| MB0277_observed | Tcf7l2 | 6ATTGA5 | 56.24795709 |
| M00691_observed | ATF1 | CYYYSACGWMA | 55.58150836 |
| M00635_observed | GT-1 | TWWWHAC | 54.59496018 |
| denovo2_observed |  | TAMAMAT | 53.96878826 |
| SW0114_observed | Exd | TGAYRD | 53.73644543 |
| MB0310_observed | Cutl1 | 5TT1AT1A5 | 53.00038847 |
| MB0334_observed | Hmbox1 | 6T1GTTA5 | 51.86847551 |
| 240_observed | BRUNOL5 | ATMCAC | 51.56453287 |
| MB0165_observed | Myb | 6AC1GTT5 | 51.01895685 |
| M01089_observed | KR | WVAAARKGTWDW | 50.85676779 |
| M00229_observed | Skn-1 | DDVATGACAWTD | 50.14281935 |
| MA0124_observed | NKX3-1 | TAARTAW | 49.45198739 |
| MA0040_observed | Foxq1 | WAWTGTTTATW | 49.14688953 |
| denovo18_observed |  | YRWWTGTRTG | 49.020141 |
| 213_observed | BRUNOL4 | WTCCAC | 48.3611912 |
| M00451_observed | Nkx3-1 | WWWTAAGTAWWT | 46.72026057 |
| denovo19_observed |  | RARARWGMRA | 46.13222519 |
| 275_observed | TPI1 | RAAAGSG | 46.06410955 |
| MB0019_observed | Yap6 | 7TTA1G2A5 | 45.89986051 |
| SW0094_observed | Lbe | YWATTA | 45.31362259 |
| MA0231_observed | lbe | YWATTA | 45.31362259 |
| M00133_observed | Tst-1 | 4T2WAWT4 | 45.18258814 |
| denovo6_observed |  | TATAVAT | 44.77480825 |
| denovo4_observed |  | CTGCTCC | 44.32814794 |
| 328_observed | NCALD | AKTARCS | 43.98969355 |
| M00998_observed | Pbx | GWTTGAWKKKWG | 43.96937247 |
| M00173_observed | AP-1 | RSTGACWMMKW | 43.92481093 |
| denovo1_observed |  | TKTWTWT | 43.62694706 |
| 8_observed | CLK1 | TGAAAG | 43.48901384 |
| MB0323_observed | Lhx3 | 6TAATT6 | 43.30193486 |
| MB0296_observed | Hic1 | 5TGCCC6 | 43.15564964 |
| M01101_observed | OVO | YAACDGYD | 41.98239486 |
| MA0051_observed | IRF2 | 2AAA4AA7 | 41.1145003 |
| M00664_observed | STE12 | ATGAAAC | 40.1771691 |
| denovo17_observed |  | YATCTCYRKC | 40.15440965 |
| M01066_observed | BLIMP1 | 4AG1G1AA3 | 40.13612861 |
| MB0330_observed | Vax2 | 4TAATT7 | 39.94698342 |
| MB0342_observed | Uncx4.1 | 6TAATT6 | 39.44959506 |
| MB0376_observed | Vsx1 | 5TAATT7 | 39.30186976 |
| M00145_observed | Brn-2 | 3AT4AAT4 | 38.89870201 |
| MB0309_observed | Hlxb9 | 6TAATT5 | 38.75980414 |
| MA0235_observed | onecut | TTGATTT | 38.75835735 |
| SW0109_observed | onecut | TTGATTT | 38.75835735 |
| SW0116_observed | Vis | TGACAG | 38.4792208 |
| MA0252_observed | vis | TGACAG | 38.4792208 |
| 355_observed | LRRFIP1 | WTSWGTAAGY | 37.94773445 |
| MA0254_observed | vvl | TKMWTR | 37.65544539 |
| M00188_observed | AP-1 | RSTGACTMMGW | 37.48799324 |
| SW0056_observed | BH1 | CRWTTAA | 36.99091419 |
| MA0168_observed | B-H1 | CRWTTAA | 36.99091419 |
| M00819_observed | Knox3 | BVKSTGACRSRK | 36.83295076 |
| SW0062_observed | Bcd | TAATCC | 36.45781651 |
| MA0212_observed | bcd | TAATCC | 36.45781651 |
| M00478_observed | Cdc5 | WTWYDTTAAAWC | 36.13326812 |
| MB0297_observed | Mybl1 | 6AC1GTT5 | 36.0094184 |
| M01108_observed | HOXA7 | MGATTGR | 35.90628149 |
| 191_observed | CKMT1B | TKTATG | 35.48804517 |
| M00403_observed | aMEF-2 | 7TWT1TA5 | 34.44428526 |
| M00172_observed | AP-1 | RSTGACWMMGW | 34.41988127 |
| 235_observed | RBM3 | TRTATG | 34.10217945 |
| 28_observed | CHES1 | CYCTGSW | 33.87501599 |
| M00018_observed | Ubx | 7TAAT1R6 | 33.54151595 |
| 17_observed | CEBPG | CTGKGA | 33.0984046 |
| 202_observed | HHAT | AGATTG | 32.41683964 |
| M00218_observed | MYB.Ph3 | 1TAAC1G6 | 32.27084423 |
| MA0068_observed | Pax4 | G24TWTT1 | 32.26530044 |
| MB0013_observed | Tbf1 | 8AGGG1T6 | 32.22306704 |
| M00771_observed | Ets | SAGGAARYGSBW | 32.06700249 |
| MB0288_observed | Zbtb7b | 3SCCCC7 | 31.63039117 |
| M00241_observed | Nkx2-5 | CAATTAWG | 31.56464565 |
| 349_observed | ZNF160 | AWCCCY | 31.42493655 |
| M00023_observed | HOXA5 (Hox-1.3) | 14AAT3G6G2 | 31.36673911 |
| MB0484_observed | Cgd2_3490 | 4GC1TGC4 | 31.36282527 |
| M00744_observed | POU1F1 | WTGWWTWWWW | 31.27368321 |
| M00777_observed | STAT | 2Y1AGAA5 | 31.20388878 |
| MA0038_observed | Gfi | CWGTGATTKR | 31.13097896 |
| MA0015_observed | Cf2_II | RYAYATATAY | 30.81987573 |
| M00087_observed | Ik-2 | RWKWRGGAAWRM | 30.52874217 |
| M00124_observed | Pbx-1b | 4T1ATTG5 | 30.16037027 |
| M00972_observed | IRF | RAAAVWGAAAV | 30.14813161 |
| 1_observed | XG | ATRMTGRMA | 30.13041213 |
| 43_observed | ZBTB46 | AATTGCT | 29.93035912 |
| MA0155_observed | INSM1 | YDMCCCCWKVMM | 29.72621399 |
| M01007_observed | SRF | 4C1A1A1A2G5 | 29.65164272 |
| M01084_observed | Antp | AAWAAMMATWAA | 29.49268623 |
| M00655_observed | PEA3 | MGGAWGT | 29.32945285 |
| 274_observed | TP73 | TTYSGC | 29.32400731 |
| M00275_observed | Mat1-Mc | 5A1CAAA7 | 29.23634893 |
| MB0413_observed | Cutl1 | 3Y1AT1AT5 | 29.06806064 |
| 290_observed | XRCC1 | AAWKTGCT | 29.04211081 |
| M01033_observed | HNF4 | TGCMCY | 28.5727553 |
| MB0400_observed | Isl2 | 4TAAK3T4 | 28.5454526 |
| SW0096_observed | Bsh | YVATTAW | 28.48606209 |

| **Haltere-specific Hth, no Ubx** |  |  |  |
| --- | --- | --- | --- |
| **Motif ID** | **factors** | **consensus** | **-10*LOG(pval)** |
| M01066_observed | BLIMP1 | 4AG1GAA4 | 61.1784443 |
| MA0114_observed | HNF4A | 4CTTTG4 | 60.6476669 |
| M00725_observed | HP1 site factor | AAWWWTYWMMAG | 57.75869414 |
| M00413_observed | AREB6 | WYWCACCTGTAC | 56.52566683 |
| denovo5_observed |  | ATAYTTA | 54.33083807 |
| M00502_observed | TEIL | AYGWAYCT | 53.07317749 |
| M00690_observed | AP-3 | WRTKKRGD | 51.26635545 |
| 48_observed | ZMAT2 | GMGGG | 50.61539191 |
| SW0005_observed | Plag-1.half.1 | GGGGSCM | 49.56857588 |
| denovo19_observed |  | TKKCTGKGGK | 48.68536953 |
| 363_observed | LHX2 | YMATTA | 47.53519394 |
| M00967_observed | HNF4, COUP | ARRGKCCAR | 47.32674066 |
| M00664_observed | STE12 | GTTTCAK | 45.54572133 |
| MA0124_observed | NKX3-1 | ATAYTTA | 44.54855425 |
| MB0101_observed | Rara | 5A1G1TCA4 | 44.4748291 |
| 249_observed | MRPL1 | ATTTCACAG | 43.49830536 |
| MB0377_observed | Nkx6-1 | 6TAATK5 | 43.40884691 |
| M00640_observed | HOXA4 | RWAAWKRG | 43.24437203 |
| MA0103_observed | ZEB1 | CACCTK | 42.16308827 |
| MB0448_observed | Nkx6-1 | 6TAAT1A5 | 41.86854666 |
| SW0111_observed | Six4 | KTMTCA | 41.74492275 |
| MA0204_observed | Six4 | KTMTCA | 41.74492275 |
| 90_observed | SSBP3 | ACMTTTCC | 41.56517883 |
| 122_observed | NR4A1 | SACMT | 41.46733193 |
| MA0222_observed | exd | WTTTGACR | 41.42380011 |
| MA0052_observed | MEF2A | CTAWAAATAR | 41.29992465 |
| 324_observed | JARID1D | MTTTKCA | 40.88282971 |
| MB0138_observed | Hnf4a | 4A2G1CCA4 | 40.88104075 |
| M00008_observed | Sp1 | KGGGCDKKGW | 40.598272 |
| MB0414_observed | Irx2 | 6ACAT1T5 | 39.75282345 |
| MB0010_observed | Ypr015c | 7ATTT1C7 | 39.44236328 |
| MB0215_observed | Nr2f2 | 4A2GG1CA4 | 39.37186979 |
| 160_observed | PAX3 | YAATKAGMSY | 38.92759739 |
| M00978_observed | LEF1, TCF1 | RRSWWCAAAGK | 38.73138887 |
| M00012_observed | CF2-II | RTATATRTR | 38.23417067 |
| MA0459_observed | tll | AAAAGYCAAM | 37.48756855 |
| SW0015_observed | tailless.new.1 | AAAAGYCAAM | 37.48756855 |
| MB0301_observed | Tcf7 | 6TTT1AW5 | 37.27453976 |
| 17_observed | CEBPG | CTGKGA | 37.0103301 |
| MB0173_observed | Lef1 | 6TTTG1T5 | 36.82301139 |
| MB0056_observed | Sut2 | 6AA1TCC8 | 36.30006955 |
| MB0233_observed | Eomes | 5TC1CAC6 | 36.29026895 |
| M01148_observed | DMRT3 | 4T1TA1C1A3 | 35.86322866 |
| M00763_observed | PPAR direct repeat 1 | 4CT1TG1C2 | 35.83546939 |
| MB0304_observed | Nkx6-3 | 5TTAAT7 | 35.82575384 |
| SW0073_observed | Dr | SCAATTA | 35.67972414 |
| MA0188_observed | Dr | SCAATTA | 35.67972414 |
| denovo1_observed |  | TKTKTWT | 35.62070607 |
| M01020_observed | TBX5 | BMRCAMCYVR | 35.50022269 |
| MA0135_observed | Lhx3 | 2ATT1A1T4 | 35.20082274 |
| 21_observed | USP39 | TTTCMRA | 35.02267116 |
| M00805_observed | LEF1 | STTTGW | 34.8215801 |
| M01107_observed | RUSH-1alpha | AWMAAWGKDW | 34.52827575 |
| M01064_observed | AGL3 | 4CYA4T1G4 | 34.17326764 |
| M00393_observed | AGL3 | 4CYA4T1G4 | 34.17326764 |
| M00083_observed | MZF1 | RGKGGGGA | 34.14769062 |
| 164_observed | EXOSC3 | TTCCA | 33.92782883 |
| 317_observed | CDK2AP1 | AATGG | 33.90643273 |
| MA0079_observed | SP1 | GGGGRMGGRG | 33.6087151 |
| MB0409_observed | Hoxa3 | 2TAATT7 | 33.60813883 |
| MB0437_observed | Hmx2 | 7AATTG5 | 33.34876076 |
| denovo13_observed |  | TBTSTKTGYK | 33.32240511 |
| MB0420_observed | Obox3 | 6AATCC6 | 33.28328033 |
| MB0454_observed | Msx2 | 7AATTA5 | 33.25987874 |
| M00451_observed | Nkx3-1 | WWWTAAGTAWWT | 33.19746435 |
| MA0109_observed | Hltf | AWMAAWGKDW | 32.77750416 |
| SW0037_observed | H20 | TTWATDR | 32.58084538 |
| MA0448_observed | H2.0 | TTWATDR | 32.58084538 |
| MB0106_observed | Zfp105 | 7ATTGA5 | 32.49335032 |
| MA0039_observed | Klf4 | DGGGYGKGGC | 32.42861188 |
| 252_observed | SRP9 | AARSTGGM | 32.26429281 |
| SW0036_observed | CG4328 | WWWATKR | 32.03248569 |
| MA0182_observed | CG4328 | WWWATKR | 32.03248569 |
| M00774_observed | NF-kappaB | 5GG1AA3C2 | 31.96967573 |
| 125_observed | HNRPH3 | TYCCAG | 31.94573532 |
| denovo2_observed |  | TATATTT | 31.87789064 |
| M01022_observed | LEF1 | BMCTTTGWWS | 31.09954158 |
| M00494_observed | STAT6 | HWWTTCHW | 30.71577138 |
| M00691_observed | ATF1 | TGWCGTSRVMG | 30.66433747 |
| denovo3_observed |  | CAGCTGC | 30.3703355 |
| 19_observed | MXD4 | AWMCCVG | 30.26088394 |
| MB0042_observed | Nhp6b | 8WATA5A2 | 30.11556824 |
| 340_observed | SUCLG1 | ATTTCWMA | 29.66484199 |
| M00407_observed | RSRFC4 | 4TA3ATA5 | 29.37897422 |
| 94_observed | VAX2 | WTTYCWMAG | 29.31312509 |
| M01061_observed | AGL2 | 4CCA1W4G4 | 28.87398183 |
| MB0453_observed | Hoxa7 | 7AATTA5 | 28.70475017 |
| MB0328_observed | Hmx1 | 6T1ATTG5 | 28.62093387 |
| M00951_observed | Grainyhead/Elf-1/NTF-1 | AAACCRGW | 28.51545353 |
| M00041_observed | ATF2:c-Jun | TGACRTKA | 27.78397561 |
| 356_observed | RPS6KA5 | GWYGTC | 27.72476729 |
| MB0285_observed | Smad3 | 6GTCTG6 | 27.33721918 |
| M00241_observed | Nkx2-5 | CWTAATTG | 27.1675296 |
| M00949_observed | AGL15 | TW1C8G1A1 | 27.09035687 |
| M01044_observed | TBX5 | CWCACACCTK | 27.00841248 |
| MB0116_observed | Gm397 | 6G1GCAC5 | 26.68012514 |
| M00489_observed | Nkx6-2 | AWWWTAAWWWWW | 26.57005438 |
| 65_observed | ESRRA | CAAGGTC | 26.54585266 |
| MA0180_observed | Vsx2 | SYAATTAAA | 26.48291837 |
| SW0014_observed | kruppel.new.4 | SRRAAGGGKKA | 26.35780704 |

| **Leg-specific Hth,**  **no Ubx** |  |  |  |
| --- | --- | --- | --- |
| **Motif ID** | **factors** | **consensus** | **-10*LOG(pval)** |
| MB0020_observed | Nhp6a | 8TAWAT8 | 413.4416 |
| MB0042_observed | Nhp6b | 2T5WATW8 | 396.2899 |
| SW0033_observed | AbdB | TTWATKW | 338.5598 |
| MA0165_observed | Abd-B | TTWATKW | 338.5598 |
| MA0124_observed | NKX3-1 | WTAYTTA | 290.9857 |
| M00138_observed | Oct1 | 8AT2AWA8 | 270.7582 |
| MB0031_observed | Sum1 | 6AW1WW2T6 | 258.5225 |
| 16_observed | HOXB13 | TTWWATGRAAA | 254.6999 |
| MA0013_observed | br_Z4 | WWRWAAAHWWW | 252.1056 |
| M00093_observed | BR-C Z3 | 4AAAC1A5 | 248.0105 |
| M00510_observed | Lhx3 | AWTTAATTWW | 244.1287 |
| MA0166_observed | Antp | TTAATKA | 240.0086 |
| SW0039_observed | Antp | TTAATKA | 237.4867 |
| MA0033_observed | FOXL1 | WWWAMATA | 232.8966 |
| SW0094_observed | Lbe | TAAYWA | 225.9967 |
| MA0231_observed | lbe | TAAYWA | 225.9967 |
| 46_observed | CSTF2 | AAATRAA | 222.4373 |
| denovo26_observed |  | 3AWA3A1A1 | 215.9535 |
| M00451_observed | Nkx3-1 | AWWTACTTAWWW | 212.5521 |
| SW0070_observed | CG33980 | TTAATTA | 207.5877 |
| MB0320_observed | Pou2f1 | 6TAATT5 | 207.5733 |
| SW0052_observed | Scr | TTAATGA | 205.0309 |
| MA0203_observed | Scr | TTAATGA | 205.0309 |
| M00101_observed | CdxA | YAKWWWW | 203.0029 |
| MA0208_observed | al | TTAATTA | 202.2413 |
| SW0067_observed | CG11294 | TTAATTA | 202.0901 |
| MA0172_observed | CG11294 | TTAATTA | 202.0901 |
| MA0236_observed | otp | TTAATKW | 198.027 |
| SW0082_observed | Otp | TTAATKW | 198.027 |
| SW0049_observed | Lab | TTAATKA | 196.4898 |
| MA0230_observed | lab | TTAATKA | 196.4898 |
| MA0170_observed | C15 | TTWAWKR | 195.4689 |
| SW0058_observed | C15 | TTWAWKR | 195.4689 |
| SW0083_observed | PdhP | WTWATTW | 192.3606 |
| MA0457_observed | PHDP | WTWATTW | 192.3606 |
| SW0080_observed | Lim1 | TAATTAA | 189.8758 |
| MA0194_observed | Lim1 | TTAATTA | 189.8758 |
| M01084_observed | Antp | AAWAAMMATWAW | 188.8849 |
| 206_observed | HDAC8 | AKAAAT | 187.3278 |
| M01098_observed | CF1A | 4AT6MWA1 | 187.0243 |
| 23_observed | PHTF1 | AAATAA | 186.9829 |
| MA0012_observed | br | WAAACWAAWWR | 184.3835 |
| MA0151_observed | ARID3A | ATHAAA | 182.5542 |
| M01011_observed | HNF1 | 5TT2T2TT7 | 180.9001 |
| SW0068_observed | CG32105 | TWWATTR | 180.6098 |
| MA0178_observed | CG32105 | TWWATTR | 180.6098 |
| MA0221_observed | eve | YTAATKA | 179.5418 |
| SW0046_observed | Eve | YTAATKA | 179.5418 |
| SW0042_observed | Btn | TTAATKW | 178.5015 |
| MA0215_observed | btn | TTAATKW | 178.5015 |
| MB0176_observed | Glis2 | 2TA5AAA2 | 177.9839 |
| MA0197_observed | Oct | TWTAATWW | 175.5484 |
| MA0192_observed | Hmx | TTAATTG | 175.5182 |
| SW0061_observed | Hmx | TTAATTG | 175.5182 |
| SW0050_observed | Lim3 | WWAATKR | 175.4022 |
| MA0195_observed | Lim3 | WWAATKR | 175.4022 |
| M00471_observed | TBP | TWTAAATW | 174.7697 |
| MB0029_observed | Smp1 | 6T2AATT8 | 172.6082 |
| SW0041_observed | Awh | YTRATTA | 170.5635 |
| MA0167_observed | Awh | YTRATTA | 170.5635 |
| 200_observed | TRMT1 | WAATGAA | 169.6717 |
| SW0053_observed | Ubx | TTWATTA | 168.0657 |
| M00094_observed | BR-C Z4 | 4KAAA3A1 | 166.3313 |
| M00096_observed | Pbx-1 | WTKWTTRWW | 166.1942 |
| M00424_observed | Nkx6-1 | 3TTAAT5 | 165.7967 |
| MA0181_observed | Vsx1 | TTAATTD | 163.4511 |
| SW0071_observed | CG4136 | TTAATTD | 163.4511 |
| 32_observed | RPL35 | TARTK | 162.4778 |
| MA0186_observed | Dfd | TTAATGA | 156.9473 |
| SW0044_observed | Dfd | TTAATGA | 156.9473 |
| SW0096_observed | Bsh | WTAAYKR | 152.6936 |
| MA0214_observed | bsh | WTAAYKR | 152.6936 |
| MB0400_observed | Isl2 | 4A3MTTA4 | 152.081 |
| MA0094_observed | Ubx | TTTAATTR | 151.969 |
| MA0135_observed | Lhx3 | 2ATT1A1T4 | 151.5909 |
| MB0449_observed | Irx4 | 5ACAT1T6 | 150.2242 |
| MB0376_observed | Vsx1 | 7AATTA5 | 147.3925 |
| MB0394_observed | Pax6 | T4AATT7 | 147.0699 |
| SW0011_observed | caudal.new.4 | TTTATTR | 146.1444 |
| MA0216_observed | cad | TTTATTR | 146.1444 |
| SW0089_observed | Unc4 | TTAATTG | 145.4447 |
| MA0250_observed | unc-4 | TTAATTG | 145.4447 |
| MA0206_observed | abd-A | TTWATTA | 145.3199 |
| SW0038_observed | Abd-A | TTWATTA | 145.3199 |
| M00790_observed | HNF1 | 3TT2T2TT6 | 144.6307 |
| MB0022_observed | Sfp1 | 7AAA2TT7 | 143.8729 |
| M00937_observed | TGA1a | RA4T8T2T3 | 140.8197 |
| MA0219_observed | ems | WWAATKR | 140.7376 |
| SW0074_observed | Ems | WWAATKR | 140.7376 |
| 256_observed | SNRP70 | ASTAAWTT | 140.5813 |
| 113_observed | GRHL1 | TDAAWCTT | 138.7296 |
| MA0240_observed | repo | TTAATTA | 137.4823 |
| SW0085_observed | Repo | TTAATTA | 137.4823 |
| M00710_observed | Zen | KTWAATRW | 136.2217 |
| MA0225_observed | ftz | TWAATKA | 135.0041 |
| SW0047_observed | Ftz | TWAATKA | 135.0041 |
| SW0059_observed | CG11085 | TTAATTG | 134.1332 |
| MA0171_observed | CG11085 | TTAATTG | 134.1332 |
| MA0179_observed | CG32532 | TTAATTK | 134.0056 |
| SW0069_observed | CG32532 | TTAATTK | 134.0056 |

| **Shared**  **(Haltere and Leg) Hth, no Ubx** |  |  |  |
| --- | --- | --- | --- |
| **Motif ID** | **factors** | **consensus** | **-10*LOG(pval)** |
| M00471_observed | TBP | TWTAAATW | 451.9864916 |
| SW0099_observed | NK71 | YWWTTAA | 421.9663023 |
| MA0196_observed | NK7.1 | YWWTTAA | 421.9663023 |
| SW0058_observed | C15 | YMWTWAA | 420.7420952 |
| MA0170_observed | C15 | YMWTWAA | 420.7420952 |
| M00101_observed | CdxA | YAKWWWW | 416.6508577 |
| M01162_observed | OG-2 | CAATTA | 389.3766439 |
| SW0036_observed | CG4328 | YMATWWW | 355.5815245 |
| MA0182_observed | CG4328 | YMATWWW | 355.5815245 |
| M00100_observed | CdxA | MTTTAWR | 343.5254582 |
| denovo2_observed |  | WAWATTT | 336.5410018 |
| SW0098_observed | Dll | HWATTAW | 331.0935071 |
| M01083_observed | Abd-A | KKAAATWWWW | 319.0408573 |
| SW0094_observed | Lbe | TWRTTA | 318.4699109 |
| MA0231_observed | lbe | TWRTTA | 318.4699109 |
| MA0063_observed | Nkx2-5 | WWMTTRW | 314.4130014 |
| denovo9_observed |  | TTAWWTW | 310.0229083 |
| 323_observed | ZRSR2 | AAMTK | 309.7807128 |
| denovo3_observed |  | ATTTATT | 309.2502466 |
| 138_observed | PHOX2A | AATTAS | 303.4395321 |
| MB0465_observed | Tlx2 | 5A1TTAA6 | 302.713893 |
| SW0060_observed | CG340131 | CAATTAA | 300.4383411 |
| MA0444_observed | CG34031 | CAATTAA | 300.4383411 |
| M01148_observed | DMRT3 | 4TGTA3A3 | 299.2583647 |
| M00489_observed | Nkx6-2 | DWWWTAAWWWTW | 295.7922389 |
| SW0083_observed | PdhP | AAATWAW | 291.4596737 |
| MA0457_observed | PHDP | AAATWAW | 291.4596737 |
| MB0042_observed | Nhp6b | 2T5WATW8 | 287.2005938 |
| SW0037_observed | H20 | YHATWAA | 276.5009181 |
| MA0448_observed | H2.0 | YHATWAA | 276.5009181 |
| M00096_observed | Pbx-1 | WWYAAWMAW | 276.355815 |
| SW0057_observed | BH2 | CAWTTAA | 276.1544309 |
| MA0169_observed | B-H2 | CAWTTAA | 276.1544309 |
| denovo5_observed |  | AWATWTY | 276.0671882 |
| SW0011_observed | caudal.new.4 | YAATAAA | 275.1983759 |
| MA0216_observed | cad | YAATAAA | 275.1983759 |
| denovo6_observed |  | ATAMTTT | 274.5639577 |
| denovo26_observed |  | 1WA3AA3T1 | 273.3909805 |
| MA0187_observed | Dll | RMAATTA | 273.0402218 |
| MA0168_observed | B-H1 | YAWTTAA | 264.8463505 |
| SW0056_observed | BH1 | YAWTTAA | 264.8463505 |
| M00713_observed | TBP | AWWTAAAWR | 256.870414 |
| M00980_observed | TBP | TTTWTAY | 255.3627233 |
| MA0033_observed | FOXL1 | TATKTWWW | 254.3879791 |
| MA0183_observed | CG7056 | YWATTMAW | 252.5537145 |
| MA0248_observed | tup | MMATTAW | 252.2551849 |
| SW0088_observed | Tup | MMATTAW | 252.2407601 |
| SW0039_observed | Antp | TMATTAA | 251.7760513 |
| 22_observed | RBBP9 | YTTTMA | 251.7237289 |
| MB0456_observed | Hlx1 | 4A1T2A1TA2 | 250.07948 |
| MA0166_observed | Antp | TMATTAA | 249.9625871 |
| MA0230_observed | lab | TMATTAA | 246.1597653 |
| SW0049_observed | Lab | TMATTAA | 246.1597653 |
| denovo13_observed |  | WWWWTATTTW | 245.8792827 |
| MA0197_observed | Oct | WAATTAWA | 244.7265992 |
| SW0033_observed | AbdB | WMATWAA | 244.27462 |
| MA0165_observed | Abd-B | WMATWAA | 244.27462 |
| SW0038_observed | Abd-A | TMATWAA | 243.9336279 |
| MA0206_observed | abd-A | TMATWAA | 243.9336279 |
| denovo27_observed |  | A5AAW2A1 | 243.3370386 |
| SW0106_observed | CG7056 | YWATTMA | 243.0782131 |
| SW0047_observed | Ftz | TMATTWA | 242.7238335 |
| MA0225_observed | ftz | TMATTWA | 242.7238335 |
| SW0034_observed | Cad | YMATWAA | 242.4463285 |
| M01011_observed | HNF1 | 7AA2A2AA5 | 239.3238937 |
| MA0094_observed | Ubx | YAATTAAA | 235.2093445 |
| M00710_observed | Zen | WYATTWAM | 235.0750618 |
| denovo33_observed |  | A1A3A4WW3 | 226.3293085 |
| MA0245_observed | slou | YHATTAA | 224.3950975 |
| SW0100_observed | Slou | YHATTAA | 224.3950975 |
| M00679_observed | Tll | AAKWYWAA | 222.3557704 |
| MB0424_observed | Dbx1 | 1W4ATTA7 | 221.2414606 |
| MA0192_observed | Hmx | CAATTAA | 221.1795534 |
| SW0061_observed | Hmx | CAATTAA | 221.1795534 |
| 200_observed | TRMT1 | WAATGAA | 220.1913466 |
| MA0240_observed | repo | TAATTAA | 220.0375978 |
| SW0085_observed | Repo | TAATTAA | 220.0375978 |
| denovo25_observed |  | 6ATTT1T1 | 218.1227439 |
| MB0121_observed | Sox21 | 4AT1AT2T4 | 218.0493252 |
| SW0050_observed | Lim3 | YMATTWW | 216.236094 |
| MA0195_observed | Lim3 | YMATTWW | 216.236094 |
| M01095_observed | AP | MMAATWHH | 215.8341256 |
| MB0029_observed | Smp1 | 8AATT2A6 | 215.069506 |
| MA0186_observed | Dfd | TCATTAA | 213.4199224 |
| SW0044_observed | Dfd | TCATTAA | 213.4199224 |
| MB0176_observed | Glis2 | 2TTT5TA2 | 212.7672064 |
| M00351_observed | GATA-3 | TWWDATCWTT | 211.7008433 |
| MA0203_observed | Scr | TCATTAA | 211.0621142 |
| SW0052_observed | Scr | TCATTAA | 211.0621142 |
| SW0105_observed | CG15696 | YAATWAA | 210.3511766 |
| 42_observed | TCEAL2 | YCATTWM | 207.716161 |
| MB0209_observed | Arid3a | 5T1AT1AA5 | 207.1282058 |
| MA0174_observed | CG42234 | YMATWWW | 204.4950522 |
| SW0035_observed | CG12361 | YMATWWW | 204.4950522 |
| MA0151_observed | ARID3A | TTTDAT | 204.4707453 |
| MA0229_observed | inv | YAATTARA | 203.7324297 |
| 345_observed | LHX4 | TCATTA | 202.7238132 |
| MA0171_observed | CG11085 | CAATTAA | 202.5727023 |
| SW0059_observed | CG11085 | CAATTAA | 202.5727023 |
| MA0037_observed | GATA3 | MWATCW | 201.641073 |
